# Progesterone receptor distribution in the human hypothalamus and its association with suicide

**DOI:** 10.1186/s40478-024-01733-y

**Published:** 2024-01-23

**Authors:** Lin Zhang, Ronald W.H. Verwer, Joop van Heerikhuize, Paul J. Lucassen, Peter W. Nathanielsz, Elly M. Hol, Eleonora Aronica, Waljit S. Dhillo, Gerben Meynen, Dick F. Swaab

**Affiliations:** 1 Neuropsychiatric Disorders Lab, Neuroimmunology Group, Netherlands Institute for Neuroscience, an Institute of the Royal Netherlands Academy of Arts and Sciences, Amsterdam, the Netherlands; 2 Brain Plasticity Group, Swammerdam Institute for Life Sciences, Faculty of Science, University of Amsterdam, Amsterdam, the Netherlands; 3 Department of Animal Science, College of Agriculture and Natural Resources, University of Wyoming, Laramie, USA; 4 Department of Translational Neuroscience, UMC Utrecht Brain Centre, University Medical Centre Utrecht, University Utrecht, Utrecht, the Netherlands; 5 Department of (Neuro) Pathology, Amsterdam UMC, University of Amsterdam, Amsterdam Neuroscience, Amsterdam, the Netherlands; 6 Department of Metabolism, Digestion and Reproduction, Faculty of Medicine, Imperial College London, London, UK; 7 Faculty of Humanities, VU University Amsterdam, Amsterdam, the Netherlands; 8 Willem Pompe Institute for Criminal Law and Criminology and Utrecht Centre for Accountability and Liability Law (UCALL), Utrecht University, Utrecht, the Netherlands; 9 Netherlands Institute for Neuroscience, Dept. Neuropsychiatric Disorders, University of Amsterdam, an Institute of the Royal Netherlands Academy of Arts and Sciences, Meibergdreef 47, 1105 BA Amsterdam, the Netherlands

## Abstract

The human hypothalamus modulates mental health by balancing interactions between hormonal fluctuations and stress responses. Stress-induced progesterone release activates progesterone receptors (PR) in the human brain and triggers alterations in neuropeptides/neurotransmitters. As recent epidemiological studies have associated peripheral progesterone levels with suicide risks in humans, we mapped PR distribution in the human hypothalamus in relation to age and sex and characterized its (co-) expression in specific cell types. The infundibular nucleus (INF) appeared to be the primary hypothalamic structure via which progesterone modulates stress-related neural circuitry. An elevation of the number of pro-opiomelanocortin^+^ (POMC, an endogenous opioid precursor) neurons in the INF, which was due to a high proportion of POMC^+^ neurons that co-expressed PR, was related to suicide in patients with mood disorders (MD). MD donors who died of legal euthanasia were for the first time enrolled in a postmortem study to investigate the molecular signatures related to fatal suicidal ideations. They had a higher proportion of PR co-expressing POMC^+^ neurons than MD patients who died naturally. This indicates that the onset of endogenous opioid activation in MD with suicide tendency may be progesterone-associated. Our findings may have implications for users of progesterone-enriched contraceptives who also have MD and suicidal tendencies.

**Supplementary Information:**

The online version contains supplementary material available at 10.1186/s40478-024-01733-y.

## Introduction

Neuroactive steroids can trigger biological functions in human hypothalamus in health and disease for instance by affecting specific stress-related neuropeptide/neurotransmitter systems. Recent studies have shown a biphasic and dynamic relationship between fluctuating progesterone levels and the risk of suicide [1, 4, 43]. Both clinical and forensic studies reported that suicidal behaviors in fertile women are more prevalent during the peri-menstrual period when peripheral progesterone levels are high [4, 43], and in menopausal and amenorrheic women when peripheral progesterone levels diminish [1]. Moreover, a nationwide prospective analysis has reported that, among contraception users, the suicide risks increase with a larger proportion of progesterone in the contraceptive [52]. In addition, higher plasma progesterone, but not estrogen levels, were found in women who had attempted suicide repeatedly [40]. Until now, however, studies on progesterone effects on the human central nervous system in relation to suicidal behaviors, were lacking [21]. In humans, progesterone regulates neural responses through the progesterone receptor (PR) [9]. A structural MRI study reported that oral contraceptive use is associated with smaller hypothalamic volumes in healthy women [12]. We therefore mapped the distribution of PR in the human hypothalamus with respect to age and sex and investigated its neurobiological signatures in glia and peptidergic neurons. Subsequently, we studied the possible relationship between changes in PR-expressing peptidergic neurons and suicide, focusing on a key stress-regulatory and PR-dense area in the hypothalamus, the infundibular nucleus (INF; in rodents homologous to the arcuate nucleus, ARC) [54].

The INF contains two distinct populations of neurons, the pro-opiomelanocortin (POMC)-expressing neurons and the neuropeptide Y (NPY)-expressing neurons, that are both involved in mood disorders (MD) and suicide. Preclinical stress studies have revealed that POMC^+^ neurons in the ARC are involved in the regulation of depressive-like behavior [46]. A combination of selective serotonin reuptake inhibitors (SSRIs) and serotonin receptor 2 A (5-HT2A) receptor antagonists has been shown to enhance therapeutic responses in a number of psychiatric disorders, while the 5-HT2A receptor is localized on ARC POMC^+^ neurons [36, 37]. Clinical studies have shown selective epigenetic alterations on the POMC gene of adolescents who suffer from depression and self-injuries [64]. Adults with major depressive disorder (MDD) have higher plasma levels of POMC, and their responses to antidepressants depend on the presence of certain POMC haplotypes [11, 29]. Moreover, naltrexone, a competitive antagonist of opioid receptors that can cause a strong increase in POMC activity in the INF, attenuates the anti-suicidality effects of ketamine [6, 62]. In postmortem observations, higher pituitary POMC expression has been found in brains of suicide completers compared to controls [30]. Thus, excessive POMC activity in the INF and pituitary seems to be related to suicidality. Contrary to POMC, rodent models of depression reported lower levels of NPY in plasma, cerebral spinal fluid, frontal-limbic regions, and ARC [15, 22, 39]. Interestingly, in these compartments, NPY levels were normalized by both, SSRIs and electroconvulsive therapy [13, 24]. Together, these preclinical observations point to marked antidepressant effects of NPY, with prominent involvement in the ARC. In humans, declining NPY expression levels in the periphery and the central limbic system were also reported in MD [27, 42, 61]. Intranasal NPY administration to patients with MDD elicits rapid antidepressant effects [38]. So far, however, the alterations in both the POMC and NPY systems in the human hypothalamus, in relation to MD and suicide, remain poorly understood.

Based on the information discussed above, we hypothesized that progesterone may play an important role in MD and suicide by affecting the activity of stress-related neurons that co-express PR and innervate their postsynaptic partners. In this study, we first investigated the distribution of PR in the human hypothalamus. In addition, we characterized PR co-expression in cell types across hypothalamic nuclei. To investigate whether PR was specially associated with MD, death ideation or suicidal behaviors, we further quantified the total numbers of POMC^+^, NPY^+^ neurons and their co-localization with PR in the INF in 28 individuals with MD and in 17 well-matched controls.

## Materials and methods

### Human brain materials

Sixty tissue blocks containing the entire hypothalamus were obtained from the Netherlands Brain Bank (NBB, Amsterdam, the Netherlands, Director: Prof. Dr. Inge Huitinga). The donor or the next of kin provided permission in advance for the autopsy, use of brain material, and clinical documentation for research. For PR mapping throughout the hypothalamus, a total of 12 control subjects (6 males and 6 females) were selected from fetal, childhood, adolescent, young, middle-aged, and elderly ages. To detect the presence of PR in early development, 3 fetal hypothalami (22, 30, and 37 gestational weeks), in addition, were included. Secondly, for observing the potential relationship between PR, MD, and suicide, 45 subjects were collected and divided into two main groups: 28 MDs and 17 matched controls (CTR) without a neuropsychiatric disorder (Table 1 and S1). Patients with MD were diagnosed according to the Diagnostic and Statistical Manual of Mental Disorder (DSM)-III, IIIR, and IV criteria by licensed psychiatrists. A neuropathologist systematically examined each brain to detect unrelated neuropathological alterations. Subjects with evidence of neurological disorders or with Alzheimer’s (Braak) stage III or higher were excluded [8]. Included subjects had no significant history of substance abuse in the 10 years before death.

**Table 1:** Demographic information

Diagnosis Cause of death	Cases, n	Psychiatric diagnosis (MDD/BD)	Reason for euthanasia application (physical diseases/MD)^1^	Age (years)^2^	Gender, male/female	PMD (hours)^2^	Brain pH^2^	Brain weight (gram)^2^
**Mood disorder**	28	22/6	-	66 (38–94)	19/9	8:55 (3:30–62:55)	6.70 (5.61–7.55)	1291 (985–1670)
Suicide (MDS)	10	9/1	-	60 (39–90)	6/4	20:35 (3:55–62:55)	6.53 (6.18–6.80)	1300 (1120–1670)
Legal euthanasia (MDE)	7	6/1	4/5^3^	61 (38–81)	5/2	6:40 (3:30 − 9:40)	6.78 (6.70–7.07)	1283 (1120–1525)
Died of natural causes (MDN)	11	7/4	-	70 (58–94)	8/3	8:55 (4:50–60:20)	6.37 (5.61–7.55)	1295 (985–1510)
**Control**	17	-	-	72 (39–88)	11/6	7:15 (3:20–41:00)	6.60 (5.37–7.28)	1259 (1115–1629)
Legal euthanasia (CE)	5	-	5/0	79 (49–83)	3/2	5:45 (3:20 − 6:30)	6.80 (6.15–7.28)	1173 (1121–1364)
Died of natural causes (CN)	12	-	-	70 (39–88)	8/4	8:30 (4:35–41:00)	6.45 (5.37–6.95)	1372 (1115–1629)

Note: 1 For practice of legal euthanasia in the Netherlands see: https://www.knmp.nl/index.php/richtlijnen/uitvoering-euthanasie-en-hulp-bij-zelfdoding. The criteria for legal euthanasia for psychiatric suffering are in principle the same as for somatic suffering. The criteria include unbearable psychological suffering and unavailable therapeutic prospects. In case of euthanasia for psychiatric reasons, an independent psychiatrist has to be consulted, regarding, among others, the patient’s decision-making competency (see Regional Euthanasia Review Committees, www.euthanasiecommissie.nl)

2 Values are provided with median within range

3 In subjects who died of legal euthanasia, two individuals applied due to a combination of physical diseases and MD

Matching for potentially confounding factors was non-parametrically tested (Table S1). These factors were age, sex, post-mortem delay (PMD), clock time of death (CTD), brain pH and brain weight (BW). It should be mentioned that the PMDs of suicide completers were estimated values. Since legal euthanasia is only performed during working hours, CTDs of the legal euthanasia groups differed significantly from the other groups. There were three sub-groups distinguished in the MD group and two sub-groups in the CTR group. The MD group contained 10 subjects with MD and completed suicide (MDS), 7 brain donors with MD and strong death ideations who died of legal euthanasia (MDE), and 11 subjects with MD who had not reported suicidality within 5 years before their natural death (MDN). Matching for the lifetime use of antidepressants among subsets (MDN, MDE and MDS) was performed according to pharmacologic effects of the medication and tested by Chi-square test. The CTR group contained 12 subjects who had no primary neurological or psychiatric disorders and died of natural causes (CN) and 5 subjects who had no primary neurological or psychiatric disorders and died of legal euthanasia due to physical diseases (CE).

Hypothalamic tissue was dissected at autopsy, fixed, dehydrated, cleared and embedded in paraffin. Coronal serial Sect. (6 μm) were cut from the level of the lamina terminalis to the mamillary bodies. Every 100th section was collected and stained with thionine for orientation.

### Immunocytochemical staining

Antibodies used in this study, their manufacturer, specificity, and staining protocol are presented in supplementary tables (Table S2 and S3). In brief, after deparaffinization and rehydration, in which sections were washed in xylene (2×10 min) and then in ethanol (100%, 100%, 96%, 90%, 70%, 50%, each×5 min), the sections were washed, treated for antigen retrieval and/or pre-incubated to block background staining. Subsequently, the sections were incubated with primary antibody, then with biotinylated secondary antibody (1:400; Vector Labs), followed by avidin-biotin-peroxidase complex (ABC; 1:800; Vector Labs). Finally, sections were incubated in a solution of 0.5 mg/mL 3, 3-diaminobenzidine (Sigma) in a total volume of 15 mL TBS containing 5 µL H_2_O_2_ 30% (Merck) and 0.035 g ammonium nickel sulfate (DAB-Ni), at room temperature for color development. For double labeling (DAB-Ni vs. DAB), ammonium nickel sulphate was not included in the second DAB development. The enzyme reaction was stopped in distilled water. Subsequently, the sections were dehydrated, cleared, and coverslipped with Entellan (Merck).

### Localization of POMC^+^ and NPY^+^ neurons

The localization of the INF was detected by performing POMC or NPY staining in the hypothalamus from the level of the optic nerves to the levels of the corpus mamillare (one section per 100th) [35]. Ten to twelve sections were stained and analyzed per hypothalamus. The fornix, optic tract and median eminence were used as anatomical landmarks. POMC^+^ neurons in the INF were labeled by an antibody directed against a major peptide, of the precursor alpha-melanocyte stimulating hormone (α-MSH) [18].

### Localization of PR/POMC^+^ and PR/NPY^+^ neurons

Double staining of PR with POMC or NPY was performed on sections adjacent to those with single POMC/NPY neuronal staining throughout the INF. In brief, sections after PR-DAB-Ni development were incubated with 20 µg/mL AffiniPure Fab fragment goat anti-rabbit IgG (H + L) (Jackson ImmunoResearch, West Grove, PA, USA) then incubated with the POMC/NPY antibody, followed by a secondary antibody and ABC incubation, and DAB development.

### Quantification of immunocytochemical staining

Four main classes of cell profiles for quantification were determined in the INF, i.e. (1) total POMC^+^ neurons, (2) nuclear PR-cytoplasmic POMC double-stained neurons (PR/POMC^+^ neurons), (3) total NPY^+^ neurons and (4) nuclear PR-cytoplasmic NPY double-stained neurons (PR/NPY^**+**^ neurons).

A 2.5× objective (Plan-Neofluar) was used on a Zeiss (Jena, Germany) Axioskop microscope mounted with a Sony (Tokyo, Japan) black/white CCD camera (model XC77CE), connecting to software package Image-Pro Plus 6.3 (Media Cybernetics Inc, Rockville, MD, USA). The total volume of the INF containing a particular cell type was calculated based upon Cavalieri principle [19]. The contour of the entire INF field occupied by the targeted neurons was manually outlined by the operator with the cursor. Subsequently, the imaging analysis system overlaid a grid of rectangular fields representing a 40× objective within the outlined cross-sectional area. To prevent double-counting, in single staining, only neurons containing a nucleolus were counted [17]. For analysis, 100% of the rectangular fields of the mask area were counted. All visible neurons with a nucleus containing a nucleolus, and within the exclusion lines, were determined using a 40× objective. In double-staining, since the nucleolus was not visible, nuclear, and cytoplasmic double-staining neurons were counted instead. By dividing the total number of cell profiles by the total volume, an estimate of the cellular density was calculated for each INF area, and the total number of the cell profiles summed up the multiplying the INF volume with the neuronal density of every level.

### Statistical analysis

S + software (version 8.2, TIBCO, Seattle, WA, USA) was used for statistical analysis. The chi-square test was used for analysis of categorical data (sex). For interval data, the Mann-Whitney test (2 samples) or the Kruskal-Wallis test with multiple comparisons (5 samples) was used [14]. In multiple testing situations the Benjamini-Hochberg correction [5] of *P*-values was applied. When the Kruskal-Wallis test was used in combination with the Benjamini-Hochberg correction, we proceeded in a two-step manner. As multiple comparisons in the Kruskal-Wallis test are only allowed if the global *P* < 0.05 [14], we first corrected the global *P*-values and then selected for further analysis only those markers for which this requirement was met. Then, the similar multiple comparisons of the allowed markers were combined and also corrected with Benjamini-Hochberg. Subsequently, the corrected multiple comparisons were recombined for each marker for presentation in the tables. Correlations were determined using Spearman’s test. CTD was also used to divide subjects into day (06:00–17:59) and night (18:00-05.59) groups. Differences in CTD and month of death (MOD, circular parameters) between controls and patients with MD were tested with the Mardia-Watson-Wheeler test [3]. For each appropriate comparison, the corresponding *P*-values were pooled and corrected according to Benjamini-Hochberg. All tests were 2-sided. Results were plotted in GraphPad Prism.

## Results

### PR distribution in the human hypothalamus in relation to age and sex

**Fig. 1:**
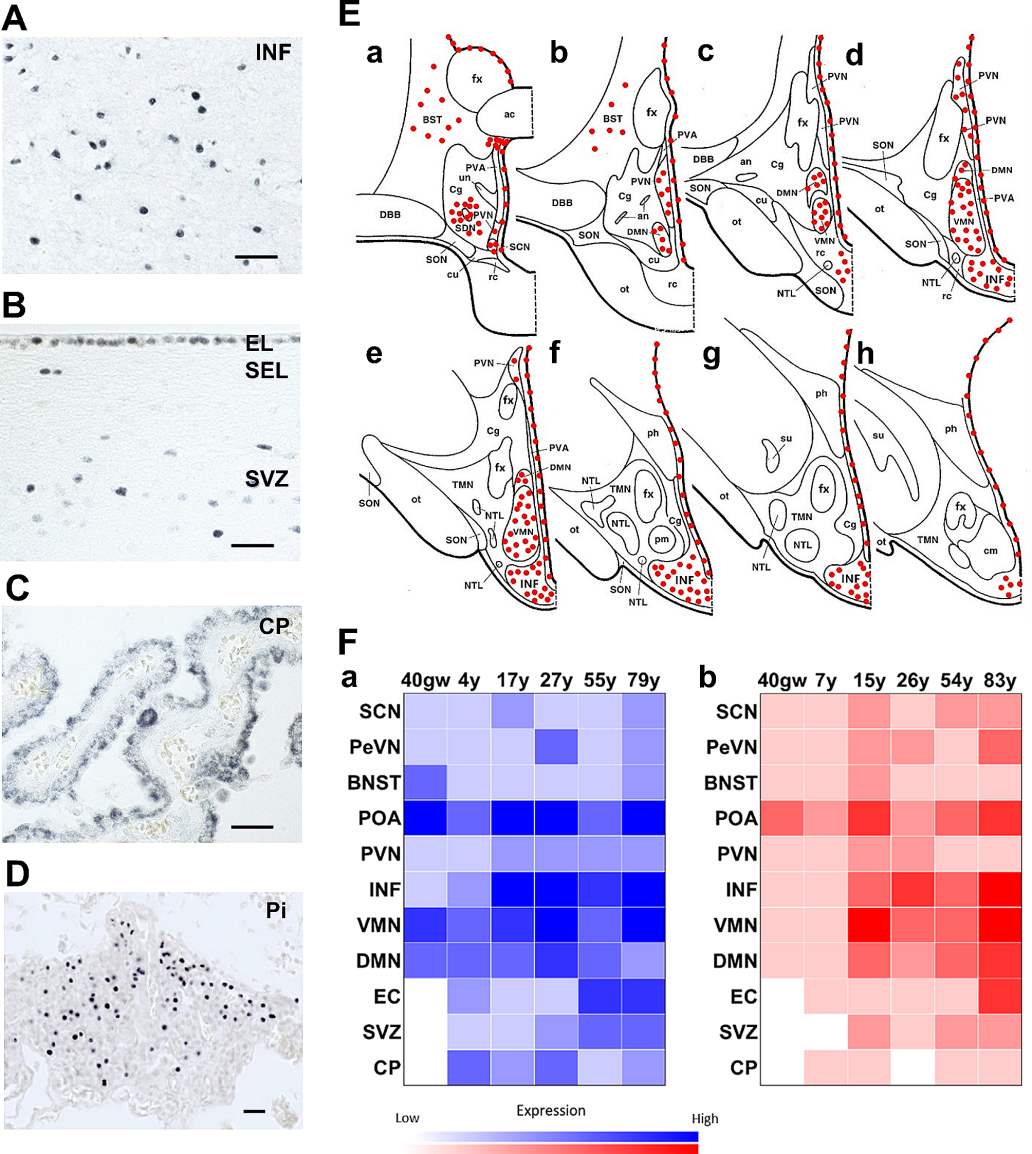
Progesterone receptor distribution in the human hypothalamus and adjacent regions. **A** Nuclear PR expression in the infundibular nucleus (INF). **B** Nuclear PR expression in cells of EL and SEL, and SVZ along lateral ventricle. **C** Cytoplasmic PR expression in the cuboidal and columnar epithelium of the choroid plexus. **D** Nuclear PR expression in the pars tuberalis (PT) of the pituitary. Scale bars: 100 μm. **E** Schematic illustration of PR mapping throughout the human hypothalamus. **F** Sex and age signature of PR-ir cells throughout human hypothalamus (*N* = 1). **a** Males (blue); **b** Females (red). Abbreviations: AC, anterior commissure; AN, accessory neurosecretory nucleus; BST/BNST, bed nucleus of the stria terminalis; Cg, chiasmatic gray; cm, corpus mamillare; CP, choroid plexus; cu, cuneate nucleus; DBB, nucleus of the diagonal band (of Broca); DMN, dorsomedial nucleus; EC, ependymal cell; EL, ependymal layer; FM, fasciculus mammillothalamicus; fx, fornix; INF, infundibular nucleus; GW, gestational weeks; NTL, lateral tuberal nucleus; ot, optic tract; PeVN, periventricular nucleus; ph, posterior hypothalamic nucleus; Pi, pituitary; pm, postero-medial nucleus; POA, preoptic area; PR, progesterone receptor; PVA, periventricular area; PVN, paraventricular nucleus; rc, retrochiasmatic nucleus; SCN, suprachiasmatic nucleus; SDN, sexually dimorphic (or intermediate) nucleus; SEL, subependymal layer; SON, supraoptic nucleus; su, subthalamic nucleus; SVZ, subventricular zone, TG, tuberal gray; TMN, tuberomammillary nucleus; un, unicate nucleus; VMN, ventromedial nucleus

From rostral to caudal in the hypothalamus and adjacent structures, cells were present with PR^+^ nuclei in the triangular septal nucleus, dorsal periventricular nucleus (DPN), anteroventral periventricular nucleus (AVPN), periventricular nucleus (PeVN), bed nucleus of the stria terminalis (BNST), suprachiasmatic nucleus (SCN), pre-optic area (POA), dorsomedial nucleus (DMN), ventromedial nucleus (VMN), PVN and INF (Fig. 1A and E and Fig. S1A and S1B). The presence of PR^+^ cells in the INF was weak at 22 gestational weeks (Fig. S1C). In addition, cells in the ependymal layer and subventricular zone (SVZ) along the lateral and third ventricle showed positive nuclear staining (Fig. 1B). In the choroid plexus (CP), both cuboidal and columnar cells showed cytoplasmic staining (Fig. 1C). Ependymal cells (EC) along the third ventricle revealed a higher density of PR expression than the lateral ventricle, which suggests stronger permeation of progesterone into the hypothalamus than the other structures. In addition, a very intense expression of PR^+^ cells were present in the pars tuberalis of the pituitary gland from the fetal period throughout the lifespan (Fig. 1D).

The intensity of PR staining in the hypothalamus across ages, in different nuclei and between sexes is depicted in Fig. 1F. We found that the presence of PR started in the fetal hypothalamus and was maintained throughout the whole lifespan. This heat map (Fig. 1F) indicates that the POA, INF, VMN and DMN revealed higher intensities of expression relative to the other positive nuclei. Surprisingly, the POA, which is functionally involved in sexual and parental behaviors in adults, showed robust PR expression in the fetal brain at the end of pregnancy. Before puberty, PR^+^ cells show stronger intensity in boys than in girls. Unexpectedly, hypothalami of elderly subjects showed overall increases in PR staining compared to the younger subjects.

Of interest, cells in the EC, SVZ and CP did not present positive signals before birth but thereafter maintained a lifelong and sex-biased expression (consistent lower intensity in females than in males). In the CP, the columnar cells in females had moderate cytoplasmic signals while both cuboidal and columnar cells in males revealed stronger expression (Fig. S1D). As columnar epithelium represents an earlier stage of CP development than cuboidal epithelium [53], we thus assume that progesterone receptor has a preference for selective expression on more immature cells.

### PR expression in hypothalamic neural progenitors and peptidergic neurons

**Fig. 2:**
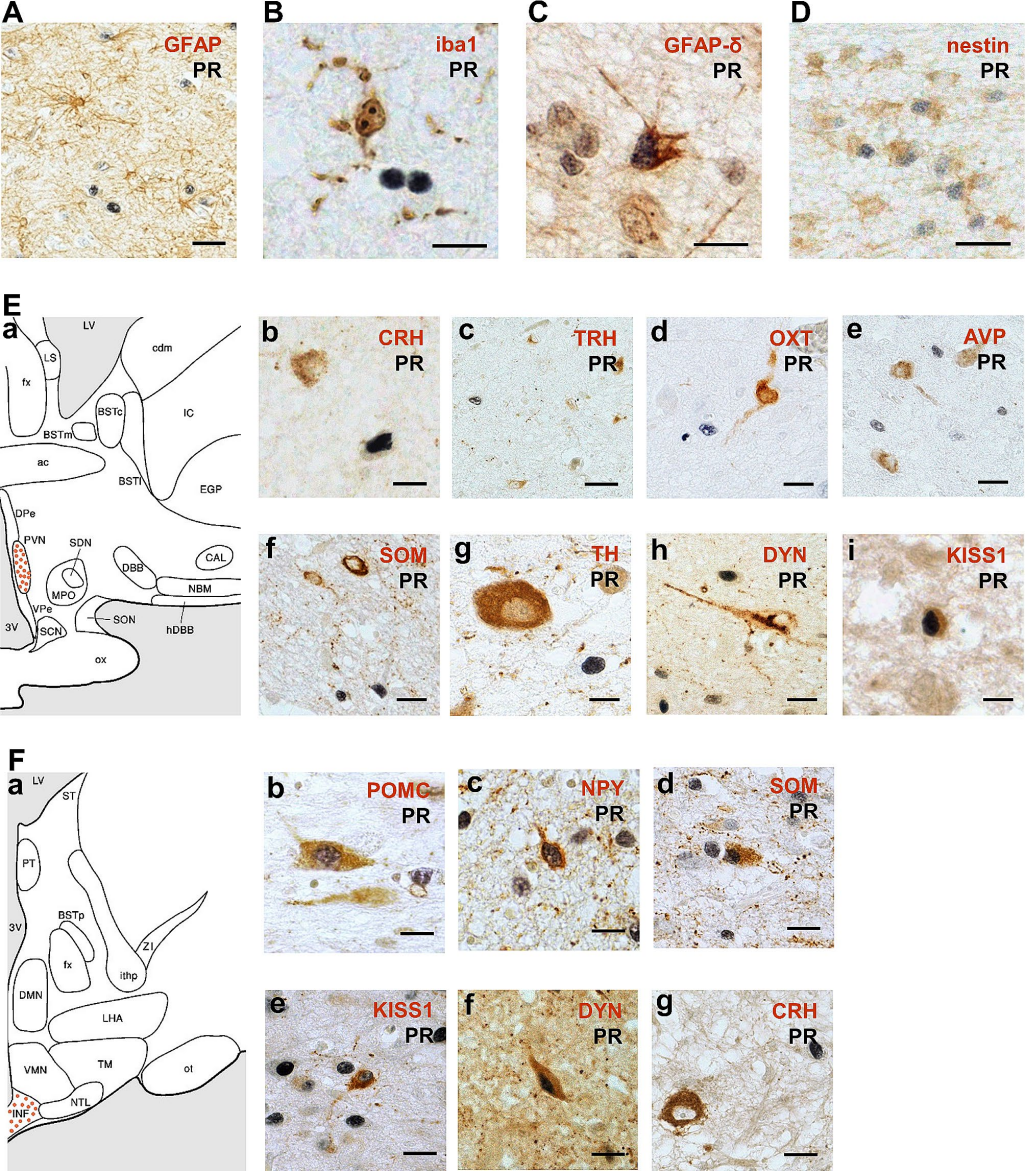
Characterization of progesterone receptor immunoreactive cells in the PVN and INF. **A** and **B** Absent co-localization of PR in astrocytes (GFAP) or microglia (iba1). **C** and **D** Co-localization of PR in neural progenitors (GFAP-δ and nestin). **E a** PVN in the human hypothalamus. **b-h** Absent expression of PR in CRH^+^, TRH^+^, OXT^+^, AVP^+^, SOM^+^, TH^+^ or DYN^+^ neurons in the PVN. **i** Co-localization of PR in KISS1^+^ neurons in the PVN. **F a** INF in the human hypothalamus. **b-f** PR expression in POMC^+^, NPY^+^, SOM^+^, KISS1^+^ and DYN^+^ neurons in the INF. **g** Absent co-localization of PR in CRH^+^ neurons in the INF. Scale bars: **A** and **D**, 50 μm; **B** and **C**, 20 μm; **E**, **a-e** 30 μm, **f** and **g** 15 μm; **F**, **a** and **b** 20 μm, **c-e** 30 μm. Abbreviations: AVP, arginine-vasopressin; CRH, corticotropin-releasing hormone; DYN, dynorphin; GFAP, glial fibrillary acidic protein; GFAP-δ, glial fibrillary acidic protein-delta; Iba1, ionized calcium binding adaptor molecule 1; KISS1, kisspeptin; NPY, neuropeptide Y; OXT, oxytocin; POMC, pro-opiomelanocortin; SOM, somatostatin; TH, tyrosine hydroxylase; TRH, thyrotropin-releasing hormone (for other abbreviations see legend Fig. 1)

We performed nuclear-cytoplasm double staining to investigate the chemical nature of PR^+^ cells in the hypothalamic nuclei mentioned in Fig. 1E and F. Using double labeling with glial fibrillary acidic protein (GFAP) or ionized calcium binding adaptor molecule 1 (iba1), we did not find PR/GFAP^+^ astrocytes or PR/iba1^+^ microglia (Fig. 2A and B). However, in the SVZ, we found PR^+^ cells co-labelled with GFAP-δ and nestin that detected neural progenitors [58] (Fig. 2C and D). The general absence of PR^+^ cells in the main white matter structures, including fornix, anterior commissure and optic nerve showed that there was no PR expression in oligodendrocytes (figures not shown).

Concerning the neuronal hypothalamic markers, double staining for PR was performed with corticotropin-releasing hormone (CRH), thyrotropin-releasing hormone (TRH), oxytocin (OXT), arginine-vasopressin (AVP), somatostatin (SOM), galanin (GAL), kisspeptin (KISS1), tyrosine hydroxylase (TH), dynorphin (DYN), POMC and NPY. In the PVN (Fig. 2E, a), we did not find PR co-expression in CRH^+^, TRH^+^, OXT^+^, AVP^+^, SOM^+^, TH^+^ or DYN^+^ neurons (Fig. 2E, b-h), but we found PR and KISS1 co-localized (PR/KISS1) neurons in elderly males (Fig. 2E, i) [56]. In the PeVN (Fig. S2A, a), PR co-labelled CRH^+^ and TH^+^ neurons (Fig. S2A, b and c), but not DYN^+^ neurons (Fig. S2A, d). In the INF (Fig. 2F, a), a considerable proportion of PR^+^ cells co-expressed POMC^+^ (PR/POMC^+^) or SOM^+^ (PR/SOM^+^) neurons (Fig. 2F, b and d) and a small proportion of NPY^+^ (PR/NPY^+^) neurons (Fig. 2F, c). Besides, we found a high proportion of PR/KISS1^+^ neurons in the INF of elderly females (Fig. 2F, e) [48]. Few DYN^+^ neurons co-labelled PR in the INF (Fig. 2F, f). Occasionally we observed CRH^+^ neurons in this region that did not co-label with PR (Fig. 2F, g). Neither did we detect PR/AVP^+^ or PR/DYN^+^ SCN neurons nor PR/GAL^+^ neurons in the sexually dimorphic nucleus (also known as the intermediate nucleus, INAH 1) (Fig. S2B and S2C).

### Elevated PR/POMC^+^ neurons contribute to the increased POMC^+^ neurons in depressed suicide patients

**Fig. 3:**
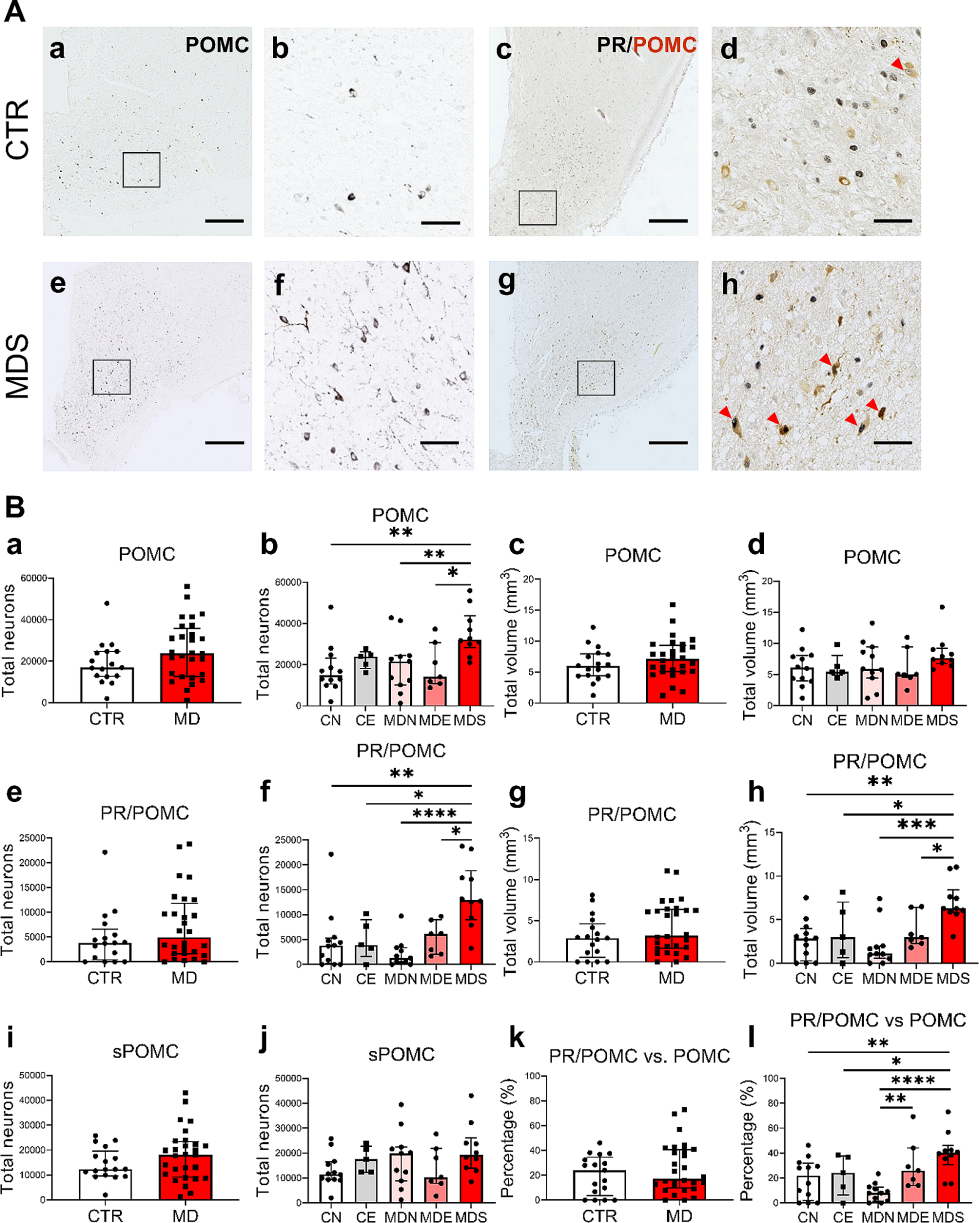
Increased numbers of PR/POMC^+^ neurons contribute considerably to the POMC^+^ neuronal increase in MDS. **A** POMC^+^ and PR/POMC^+^ neurons (red arrows) in the INF of a control subject and a patient with MD who died of suicide. Higher magnification of the areas framed in **a, c, e** and **g** are shown in **b, d, f** and **h**, respectively. **B** Analyses between CTR and MD subjects (two-group comparison), and their subsets (five-group comparison) on: total neurons and volume of POMC (**a-d**), PR/POMC (**e-h**), total neurons of sPOMC (**i-j**), proportion of PR/POMC neurons in POMC neurons (**k-l**). Note that patients with MD who died of suicides or legal euthanasia presented more PR/POMC^+^ neurons in the INF than patients who died naturally. Scale bars: **a**, **c**, **e** and **g**, 1 mm; **b**, **d**, **f** and **h**, 100 μm. Abbreviations: CE, control subjects who died of legal euthanasia; CN, control subjects who died of natural causes; CTR, control subjects; MD, mood disorders; MDE, subjects with mood disorders who died of legal euthanasia; MDN, subjects with mood disorders who died of natural causes; MDS, subjects with mood disorders who died of suicide; PR/POMC, neurons co-labeling progesterone receptor and pro-opiomelanocortin; sPOMC, pro-opiomelanocortin neurons that did not express progesterone receptor. Note: * indicates 0.01 ≤ *P*<0.05, ** indicates 0.001 ≤ *P*<0.01, *** indicates 0.0001 ≤ *P*<0.001, **** indicates 0.00001 ≤ *P*<0.0001, Global-*P*<0.05

We quantified the total number of POMC^+^ and PR/POMC^+^ neurons in the INF. No difference was observed between MD patients and controls (Fig. 3B, a and c). Of note, some POMC^+^ neurons with two nucleoli were present in both MD and CTR (also in SOM-ir neurons, Fig. S3, A and B). The number of PR and POMC double-staining neurons was determined on adjacent sections but did not differ between MD patients and controls (Fig. 3B, e and g). Such PR/POMC^+^ neurons were also observed in the INF of a 4-year-old child (Fig. S3C). The number of POMC^+^ neurons that did not co-localize PR (sPOMC^+^ neurons) was not different between MD and controls (Fig. 3B, i).

In a subset analysis, however, a robust increase in the total number of POMC^+^ neurons was observed in depressed suicide cases (MDS) compared to the other MD subsets and CN subjects (Fig. 3B, b). Additionally noteworthy is that POMC^+^ neurons in the controls who died of legal euthanasia did not reveal alterations relative to the MDS and CN groups, but its median value was in between, suggesting some potential increase of POMC^+^ neurons in subjects with death ideation. Total volumes of the POMC^+^ neuron cluster were not different within the five subgroups (Fig. 3B, d), indicating that, in brains of those who completed suicide, an elevation of POMC^+^ neurons are the major argument to explain the higher POMC^+^ density in these volumetrically stable INFs.

By analysing PR/POMC^+^ neuron numbers as well as volumes, we observed an exclusive increase in MDS relative to the other subgroups (Fig. 3B, f and h), indicating that increased PR/POMC^+^ neurons were recruited from the adjacent area within the INF. sPOMC^+^ neurons remained unchanged within the five groups (Fig. 3B, j). In addition, a higher proportion of POMC^+^ neurons that co-labelled PR was also detected in MDE versus MDN (Fig. 3B, l). It suggests that in individuals with MD, PR/POMC^+^ neurons may be related to suicidality, including suicidal behaviours and even sensitivity to suicide ideation. We concluded that the elevated sum of POMC^+^ neurons in the INF of suicidal cases was due to an increased number of PR/POMC^+^ neurons.

### PR is not associated with a reduced NPY expression in depression

**Fig. 4:**
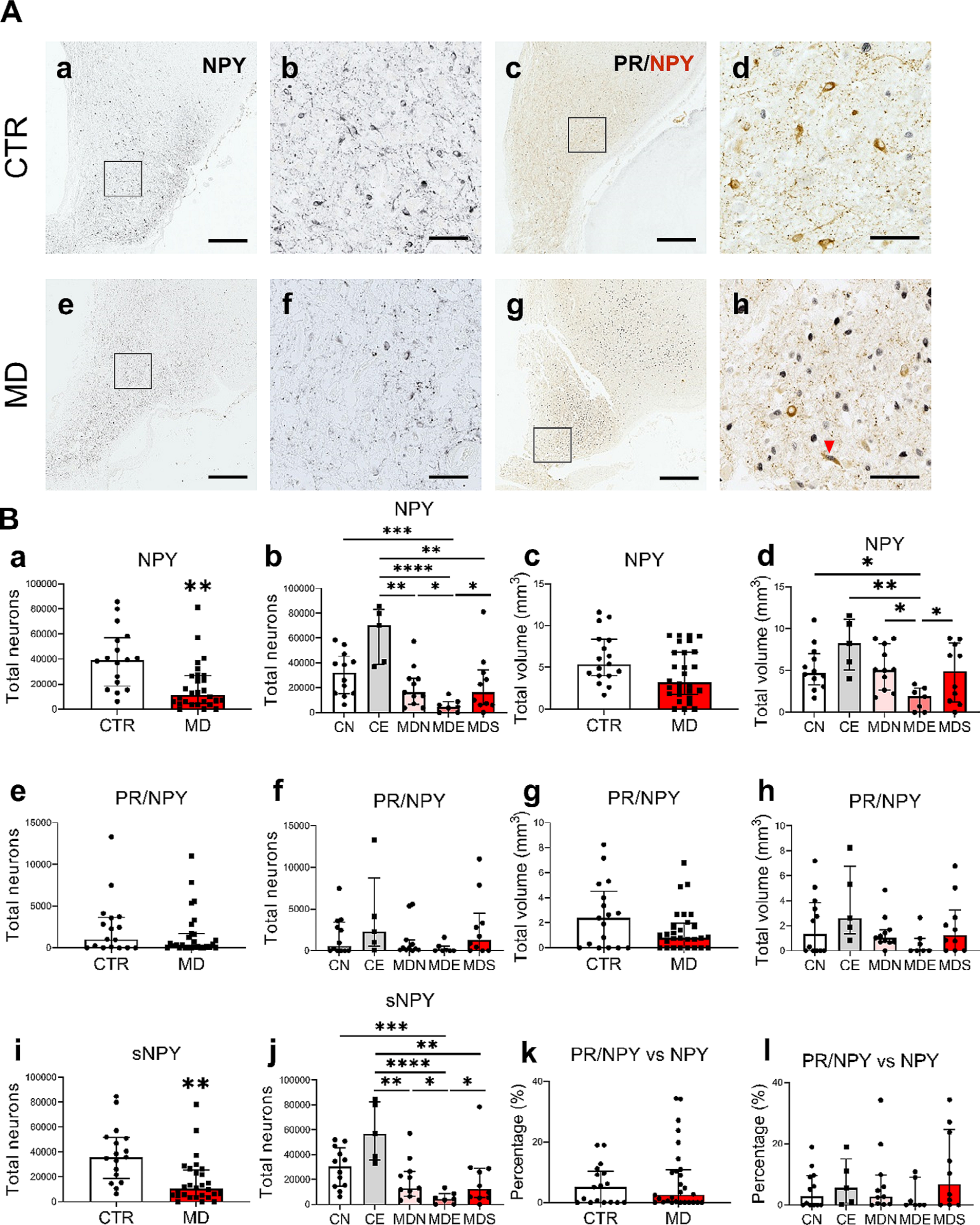
Reduction of total number of NPY^+^ neurons in the INF of MD is not associated with PR. **A** NPY^+^ and PR/NPY^+^ neurons (red arrows) in the INF of a control subject and an individual with MD. Higher magnification of the areas framed in **a, c, e** and **g** are shown in **b, d, f** and **h**, respectively. **B** Analyses between CTR and MD subjects, and their subsets on: total neurons and volume of NPY (**a-d**), PR/NPY (**e-h**), total neurons of sNPY (**i-j**) and the proportion of PR/NPY neurons in NPY neurons (**k-l**). Note the sharp drop of NPY expression in MDE compared to CE (b, j). Scale bars: **a, c, e** and **g**, 1 mm; **b, d, f** and **h**, 100 μm. Abbreviations: PR/NPY, neurons co-labeling progesterone receptor and neuropeptide Y; sNPY, neuropeptide Y neurons that did not express progesterone receptor. Note: * indicates 0.01 ≤ *P*<0.05, ** indicates 0.001 ≤ *P*<0.01, *** indicates 0.0001 ≤ *P*<0.001, **** indicates 0.00001 ≤ *P*<0.0001, Global-*P*<0.05

A marked reduction of the total number of NPY^+^ neurons was found in subjects with MD (Fig. 4B, a and c). Subset analysis showed that individuals with MD who died of legal euthanasia had the lowest NPY^+^ neuronal counts among the five subgroups (Fig. 4B, b and d). Strikingly, control subjects who died of legal euthanasia presented the highest NPY expression, in sharp contrast to MDE subjects, which indicates that NPY expression in the INF is seriously inhibited in MD.

The number of PR/NPY^+^ neurons remained unchanged in both two- and five-group comparisons (Fig. 4B, e-h). Similar changes with total NPY^+^ neurons have been found in non-PR-expressing NPY^+^ (sNPY^+^) neurons (Fig. 4B, i and j), suggesting that PR may be unrelated to these decreases. In contrast to the high proportion of POMC^+^ neurons that co-expressed PR, NPY^+^ neurons that showed co-localization with PR are generally less than 10% of the total number of NPY^+^ neurons (Fig. 4B, k and l). Of note, the reduced number of NPY^+^ neurons in the INF of depressed patients was not affected by their psychiatric diagnosis (MDD vs. BD).

### Reciprocal innervations between POMC, NPY, CRH and TRH

To observe possible interactions between POMC^+^ and NPY^+^ neurons in the INF, and CRH^+^ and TRH^+^ neurons in the PVN, we performed double staining for these peptides to reveal their morphology and the distribution of neuronal elements. As shown in Fig. S4, the cell body of POMC^+^ and NPY^+^ neurons, CRH^+^ and TRH^+^ neurons were located in the INF and PVN respectively, while their axonal varicosities were distributed in both nuclei. We observed NPY^+^, CRH^+^ and TRH^+^ neurons that received considerable POMC^+^ contacts on both perikarya and dendrites, while NPY^+^ terminals densely targeted POMC^+^, CRH^+^ and TRH^+^ neurons with well-defined juxtapositions. Similarly, both CRH^+^ and TRH^+^ fiber varicosities were surrounding POMC^+^/NPY^+^ neurons with multiple contacts.

### Confounder analysis

We included age, sex, CTD, BW, and brain pH in the confounding analysis of our results. There were no differences in the NPY expression between sexes within MD and CTR separately, but we found a sharp reduction of NPY^+^ and sNPY^+^ neurons in MD versus CTR, which was significant only in males (Fig. S5A). POMC expression did not reveal sex-preferred alterations (Fig. S5B). Since we have matched our (sub)groups for these factors, the observed correlations did not affect our conclusions.

## Discussion

Human hypothalamus plays a major role in mood and stress modulation, but the hypothalamic pathophysiologic systems that differentiate suicidality from MD are poorly understood and continue to be debated. Here, we show for the first time, strong PR-associated changes in a specific group of peptidergic neurons in relation to suicide. Our major findings are: (1) hypothalamic PR-containing cells are concentrated in the nuclei adjacent to the third ventricle in a relatively stable pattern in both sexes throughout the lifespan, (2) various peptidergic neurons as well as neural progenitors co-express PR, (3) POMC^+^ neurons in the INF are increased in suicide completers with MD, and this elevation is primarily due to PR/POMC co-expressing neurons, (4) NPY^+^ neuron number in the INF is sharply reduced in patients with MD, but PR/NPY co-expressing neurons do not participate in this decrease. We also report a sex difference of a reduced NPY expression in males but not females, when comparing MD cases versus controls. Together, these findings add to the growing evidence supporting the involvement of endogenous opioid system in suicide and suggest that progesterone-mediated, hypothalamic peptide alterations are an important molecular basis of psychopathology in suicide.

Local PR expression in human hypothalamus has been reported [26, 32]. In this study, we show the distribution of PR^+^ cells throughout lifespan in the human hypothalamus, which is generally in accordance with animal studies [59]. Here, PR was present in specific hypothalamic nuclei known to have diverse hormone-mediating and neurodevelopmental functions in, e.g. the anxiety and stress response, neurogenesis, sexually dimorphic behaviors, circadian rhythm, feeding and metabolism, drinking, parental behavior and sleep [55]. This widespread distribution suggests that progesterone via hypothalamic PR may affect a multitude of neuroendocrine regulators. The largely identical distribution pattern over the ages between sexes thereby seems to be independent of the progesterone plasma levels. Central progesterone could be derived from progesterone entering the brain after being synthesized in the periphery as a sex hormone, or it could be locally produced by astrocytes as a brain-derived neuroactive steroid [49]. Of note, the density of PR^+^ cells along the third ventricle decreased from the ventromedial to the dorsolateral hypothalamus, indicating that the INF, a nucleus that is anatomically located outside the blood-brain barrier, might be a major structure through which peripheral progesterone enters the brain. Positive PR expression in the epithelial cytoplasm, but not in the nuclei of CP, supports this possibility, which is also in accordance with the idea that both peripherally synthesized and neuroactive progesterone inputs may regulate emotional and metabolic integration [34].

The presence of PR in neural progenitors and specific populations of peptidergic neurons potentially determines the neurobiological substrate that is modulated by progesterone in the human hypothalamus. Selective co-expression between PR and neuropeptides, such as CRH, SOM and TH, in different human hypothalamic nuclei indicates that progesterone regulation of the neuroendocrine system has region- and function-heterogeneous targets. We found PR to be present from early in the second trimester of pregnancy until old age. PR further presented co-localization with neural progenitors in the SVZ, which is the main neurogenic niche in the adult human brain [58], indicating that progesterone activity may be involved in human adult neurogenesis [10]. Supporting this possibility, we reported here binuclear POMC^+^ and SOM^+^ neurons that may correspond to the late phase of mitosis and post-mitotic maturation of neurogenesis in the INF.

Studies have demonstrated that endogenous opioids play important roles in modulating stress-related behaviors [2, 41]. Although POMC^+^ neurons in the INF are well known to be involved in eating behavior, the elevated POMC expression we describe here, indicates the involvement of the endogenous opioid system activation in suicide [62]. Progesterone is increased following stress [28]. In ovariectomized monkeys, it has been shown that progesterone administration causes the release of hypothalamic β-endorphin, a major derivative of POMC, into peripheral blood [60]. Evidence has shown that the plasma level of β-endorphin was increased in suicide attempters, while this increase positively correlated with lifetime suicide attempts [7, 20, 25]. One may assume that in individuals experiencing suicidality, progesterone may modulate POMC synthesis, leading to opioid activation and subsequent suicidal behaviors [33, 63]. Among subjects with MD, the numbers of POMC^+^ and PR/POMC^+^ neurons both increased in suicide, and the proportion of PR/POMC^+^ neurons increased both in MDS and MDE. This indicates that progesterone is associated with suicide ideations, but the final and fatal suicidal behaviors, which are absent in patients with legal euthanasia, may have different molecular bases. In addition, an animal study reported that progesterone administration can elicit long term POMC elevations [44], in spite of the fact that the elimination half-life of progesterone by oral intake was approximately 5 h [45]. This may provide a possible explanation for the observation that women have a high tendency of suicide during menstrual and postpartum periods when their peripheral progesterone levels drop sharply [1, 4, 43]. Moreover, PR expression has been found to be stronger in both sexes during adolescence than during childhood, and may correspond to an increased suicide rate in adolescents [47].

In this study, the expression of NPY in the INF of depression cohorts was sharply reduced, agreeing with a previous report that showed decreased expression of NPY in the prefrontal cortex and hippocampus of depressed suicides [50]. Our earlier research showed that the duration of an agonal state before death is accompanied by a decreased NPY expression [16]. For this reason, we studied brain donors who underwent legal euthanasia as they have very short agonal states. By comparing NPY^+^ neurons in CE versus MDE and CN, we found that a reduction in numbers was present only in MD. Our data agreed with an antidepressant effect of NPY, and we concluded that MD, rather than death ideations, could play a major role in impairing hypothalamic NPY reserves. It is not known whether the increase of NPY in the plasma of suicide completers is caused by the NPY released from other regions of the brain or from other organs [31]. Given that there is a small proportion of PR/NPY^+^ neurons that showed no changes in the different conditions, we have no evidence to connect their association with MD or suicidality.

Neurons in the INF control behaviors by synaptic regulations across various neural circuits. We investigated a first and important step in this network, i.e., the relationship between the INF and PVN. The observations that POMC^+^, NPY^+^, CRH^+^ and TRH^+^ neurons terminate on each other provide a morphological substrate for their reciprocal interaction. Terminals of POMC^+^ neuron axon projections, including the other hypothalamic nuclei, thalamus, amygdala and brainstem, could be the targeted regions that respond to adverse stimuli. Among them, the ventral tegmental area and thalamus have been associated with the opioid system in depression [33, 46]. Tracing the INF projections of this opioid population may help to identify brain areas interconnected to the neuropathology of death ideation and suicidal behaviors.

It is important to note that only MD males, but not MD females have fewer NPY^+^ neurons in the INF. To the best of our knowledge, studies that showed hypothalamic NPY reductions in depression models and normalization of depressive-like behaviors after treatments used only male animals [15, 23, 24]. Previous research showed that NPY expression in the INF is activated by testosterone only in male animals [57] and testosterone deficiency is accompanied by a higher incidence of depression, especially in males after midlife [51]. This may relate to the less robust reductions in NPY^+^ neurons in females. As the subjects in this study have a median age over 60, decreases in testosterone possibly underlie the observed NPY reduction.

There are a few aspects of our study that require consideration. Menstrual information was not recorded in primary clinical data. Since the number of younger women is small, this will likely not influence our general conclusions. Secondly, the sample size is relatively small, limited by the unique cases of legal euthanasia and the inclusion and exclusion criteria we used. Another limitation is that we could not stratify the manner in which every subject accomplished suicide. For instance, information about violent or non-violent categories was not available in the clinical data. It prevented us from analyzing whether the suicide manner is confounding the progesterone-associated activation of the opioid system. In addition, our study employed human postmortem brain tissue which makes it inevitable to consider the possible role of antidepressants. Therefore, we distributed the antidepressant users, in relation to the pharmacologic effects in MD, equally among the subsets.

In conclusion, we systematically examined the anatomical and functional organization of PR in the human hypothalamus and indicated its association with suicide by demonstrating increased numbers of PR/POMC^+^ neurons in the INF in patients with MD and suicidality. Moreover, the identification of distinct neuronal subpopulations involved in the effects of progesterone on suicidality will help better understand the molecular mechanisms underlying MD and suicide. We suggest that special attention should be paid to females with MD, who use hormonal contraception containing a high concentration of progesterone.

### Electronic supplementary material

Below is the link to the electronic supplementary material.

Supplementary Material 1

Supplementary Material 2

Supplementary Material 3

Supplementary Material 4

Supplementary Material 5

Supplementary Material 6

Supplementary Material 7

## Data Availability

The datasets during and/or analyzed during the current study available from the corresponding author on reasonable request.
