# Supplementary figures and images for: Progesterone receptor distribution in the human hypothalamus and its association with suicide
Source: Acta Neuropathol Commun. 2024 Jan 23;12:16. doi: 10.1186/s40478-024-01733-y (PMC10807127; doi:10.1186/s40478-024-01733-y)

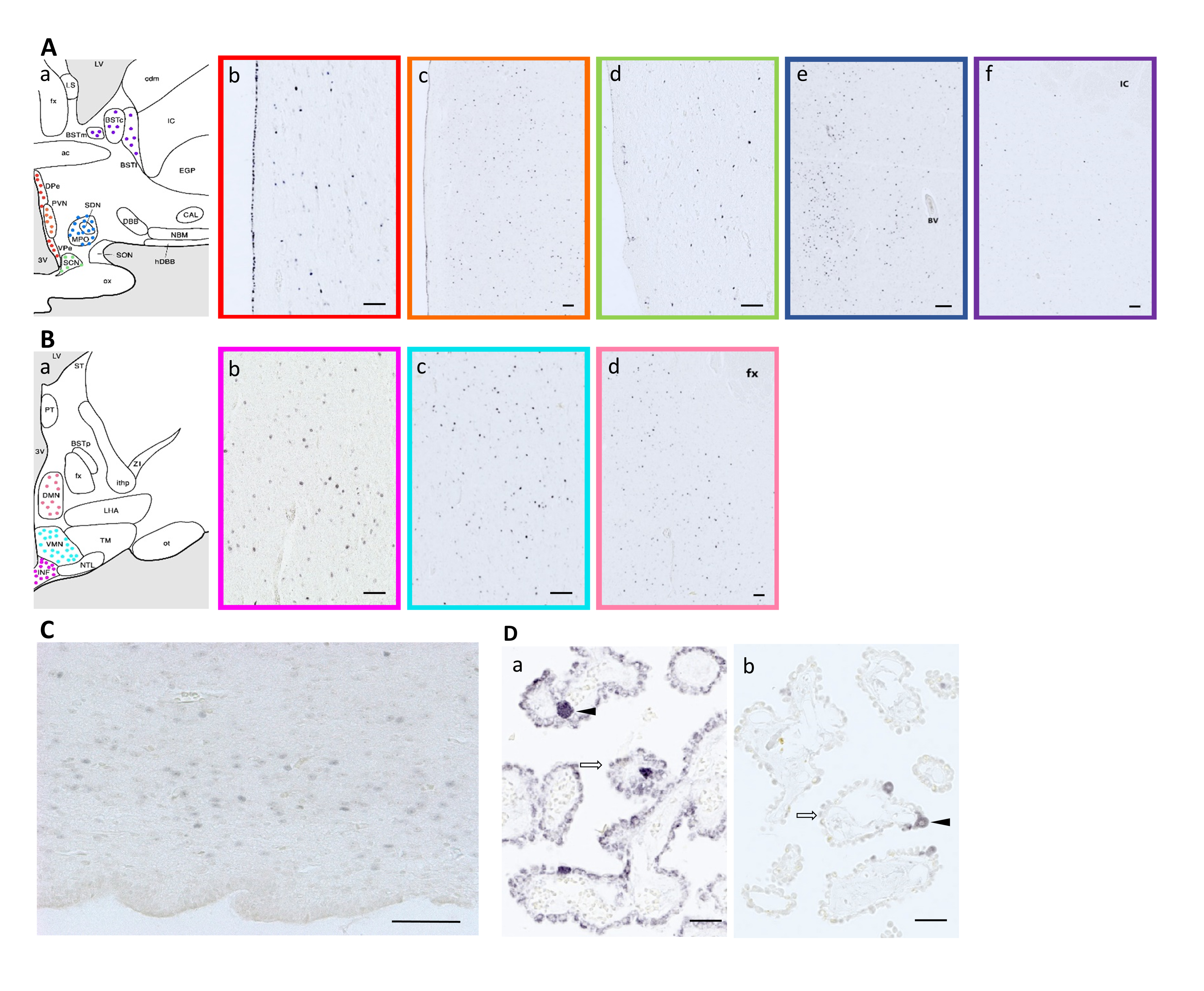

Supplement: Supplementary file 1 — Supplementary Material 1 [file 40478_2024_1733_MOESM1_ESM.tif]

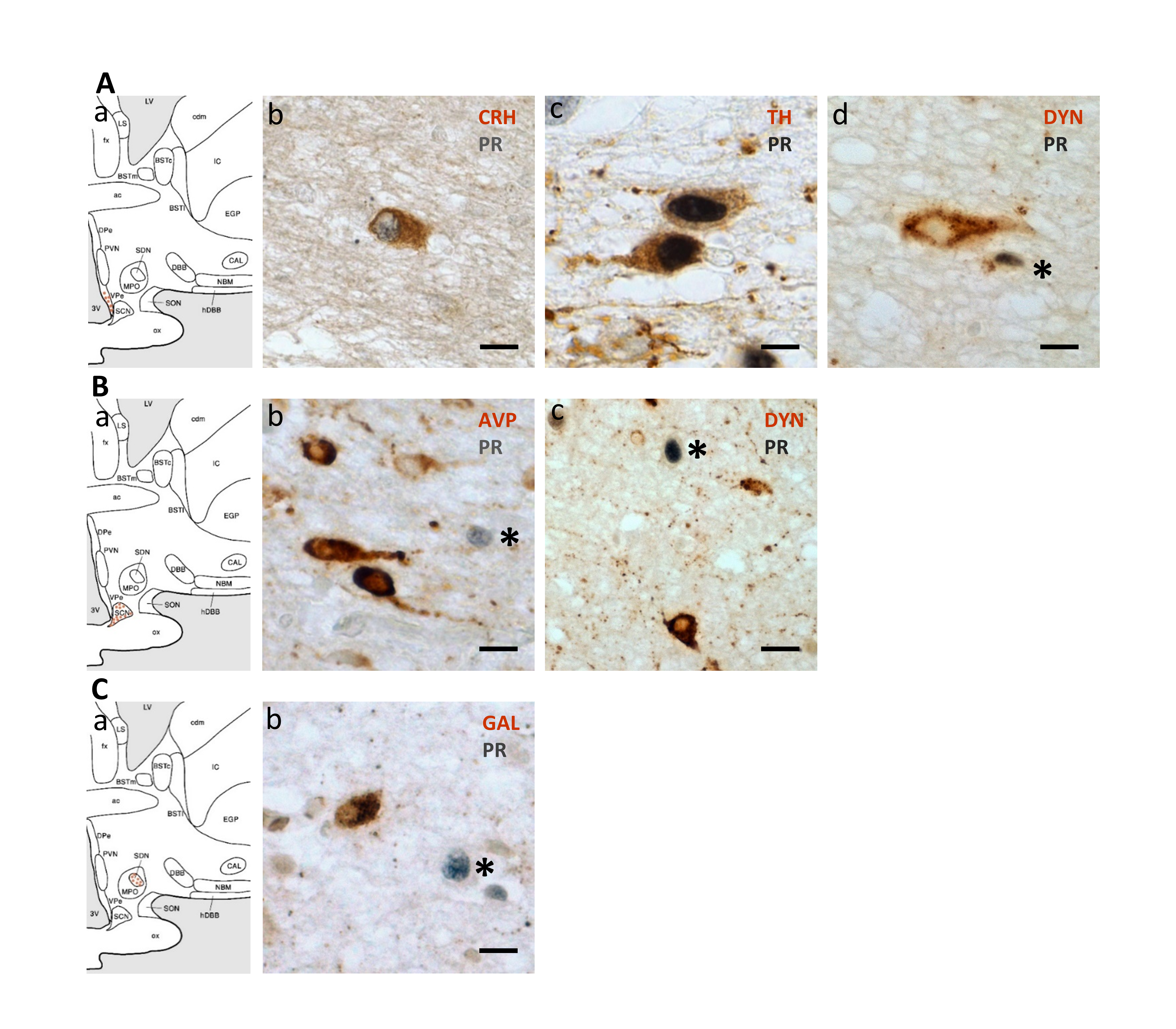

Supplement: Supplementary file 2 — Supplementary Material 2 [file 40478_2024_1733_MOESM2_ESM.tif]

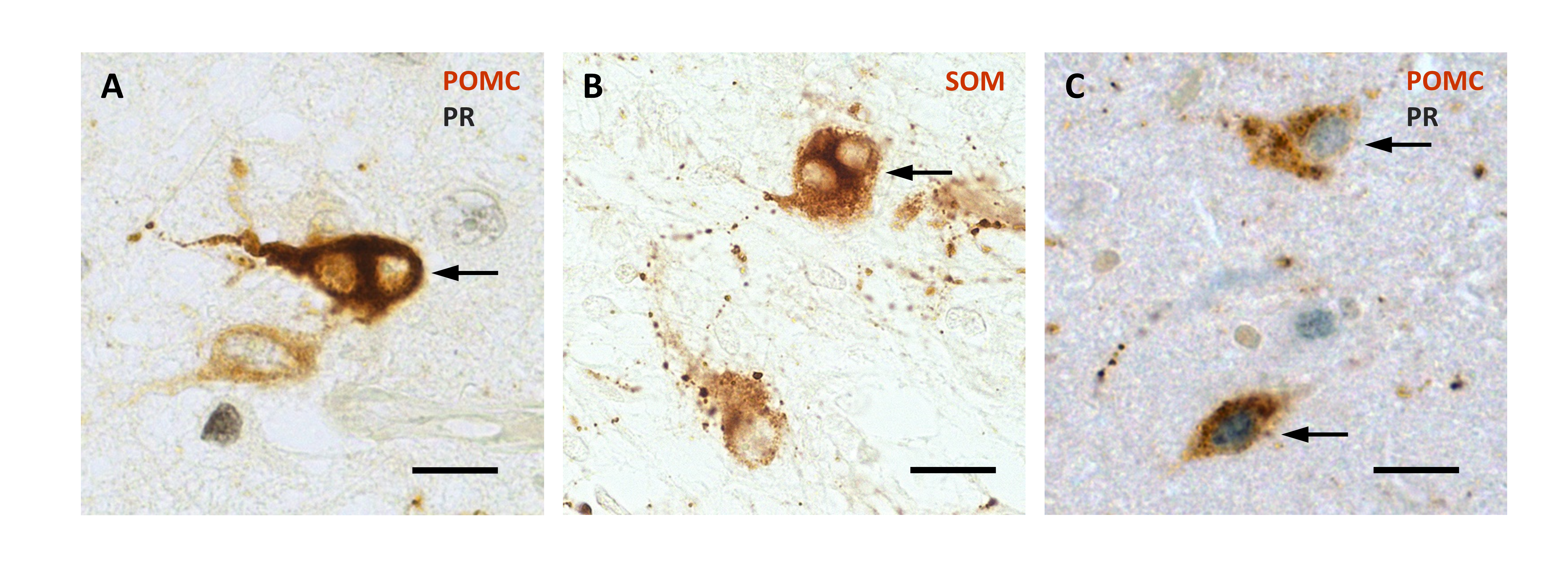

Supplement: Supplementary file 3 — Supplementary Material 3 [file 40478_2024_1733_MOESM3_ESM.tif]

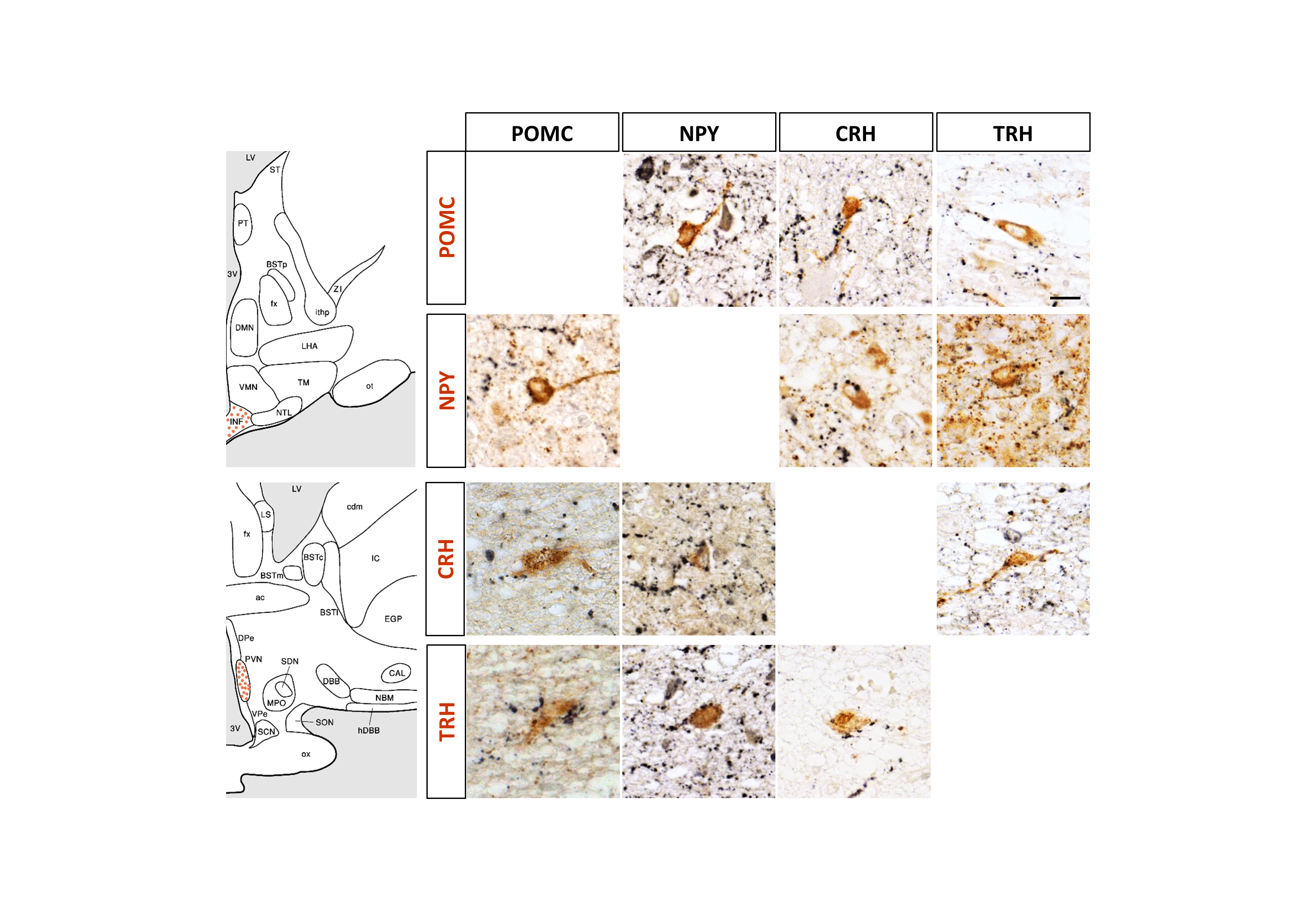

Supplement: Supplementary file 4 — Supplementary Material 4 [file 40478_2024_1733_MOESM4_ESM.tif]

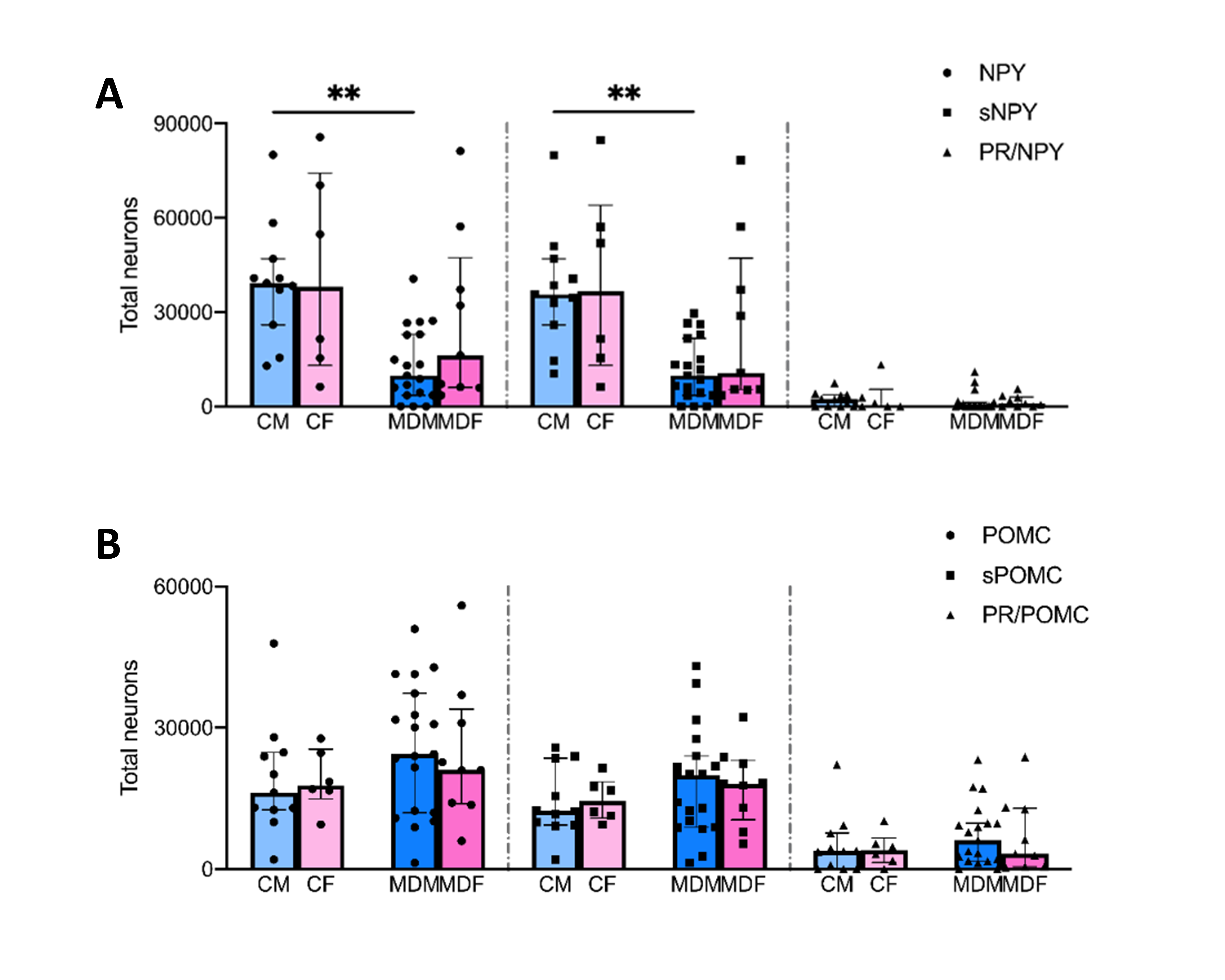

Supplement: Supplementary file 5 — Supplementary Material 5 [file 40478_2024_1733_MOESM5_ESM.tif]
