# Supplementary material for: Progesterone receptor distribution in the human hypothalamus and its association with suicide
Source: Acta Neuropathol Commun. 2024 Jan 23;12:16. doi: 10.1186/s40478-024-01733-y (PMC10807127; doi:10.1186/s40478-024-01733-y)
Supplement: Supplementary file 6 — Supplementary Material 6 [file 40478_2024_1733_MOESM6_ESM.docx]

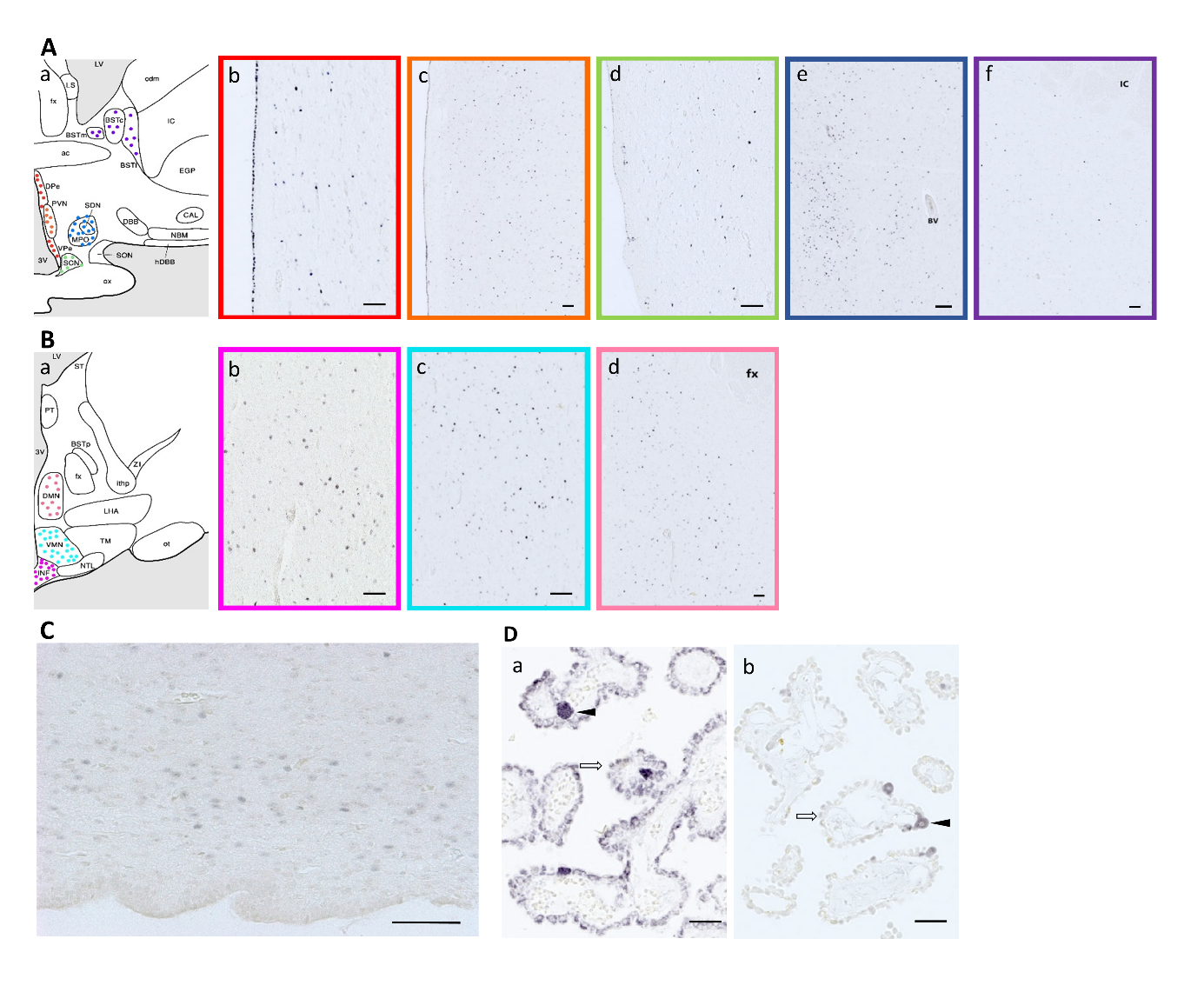


**Figure S1 Progesterone receptor in human hypothalamic nuclei. A** Nuclear PR expression in the anterior hypothalamus. Higher magnification of the areas plotted in schematic figure **a** are shown in **b-f**. Colors of the dots correspond to the frames. **B** Nuclear PR expression in the posterior hypothalamus. Higher magnification of the areas plotted in schematic figure **a** are shown in **b-d**. Colors of the dots correspond to the frames. **C** Nuclear PR expression in the infundibular nucleus of 22 gestational weeks. **D** Cytoplasmic PR expression in the CP between a control male (**a**) and a control female (**b**). Empty arrows indicate the cuboidal epithelium while solid arrowheads show the columnar epithelium. Scale bars: 100 μm. Abbreviations: 3V, third ventricle; ac, anterior commissure; BSTc, central nucleus of the bed nucleus of the stria terminalis; BSTl, lateral nucleus of the bed nucleus of the stria terminalis; BSTm, medial nucleus of the bed nucleus of the stria terminalis; BSTp, posterior nucleus of the bed nucleus of the stria terminalis; BV, blood vessel; CAL, accumbens nucleus, lateral part; cdm, medial caudate nucleus; CP, choroid plexus; DBB, diagonal band of Broca; DMN, dorsomedial nucleus; DPe, dorsal periventricular nucleus; EGP, external globus pallidus; fx, fornix; hDBB, horizontal limb of the diagonal band of Broca; IC, internal capsule; INF, infundibular nucleus; ithp, inferior thalamic peduncle; LHA, lateral hypothalamic area, LS, lateral septum; LV, lateral ventricle; MPO, medial preoptic area; NBM, nucleus basalis of Meynert; NTL, nucleus tuberalis lateralis; ot, optic tract; ox, optic chiasm; PR, progesterone receptor; PT, paratenial thalamic nucleus; PVN, paraventricular nucleus; SCN, suprachiasmatic nucleus; SDN, sexually dimorphic (or intermediate) nucleus; SON, supraoptic nucleus; ST, stria terminalis; TM, tuberomammillary hypothalamic nucleus; VMN, ventromedial nucleus; VPe, ventral periventricular nucleus; ZI, zona incerta.


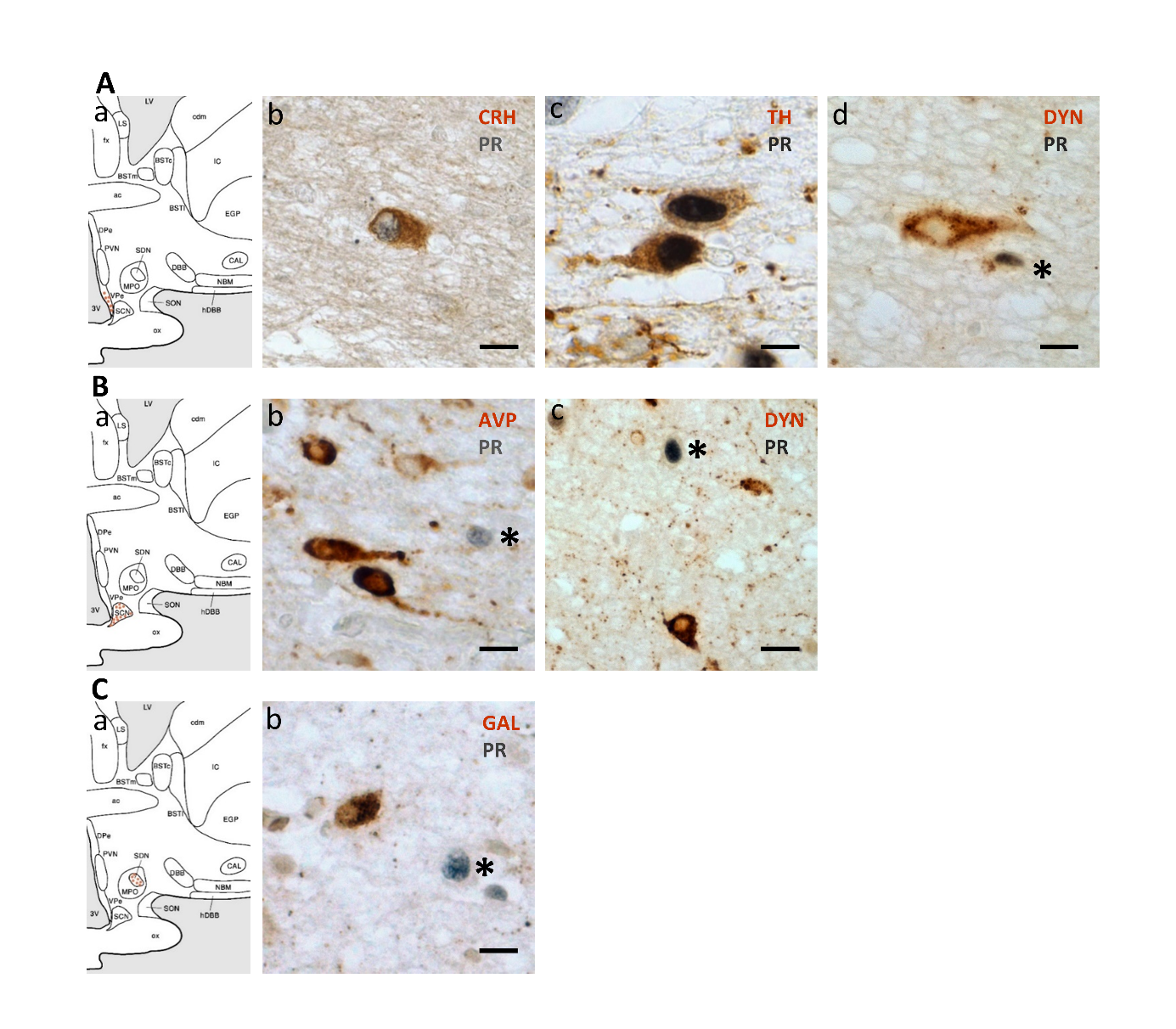


**Figure S2 Double staining of PR^+^ cells with different neuropeptides in the ventral periventricular nucleus (VPe), suprachiasmatic nucleus (SCN) and sexually dimorphic nucleus SDN). A a** VPe in the human hypothalamus. PR**^+^** cells in the VPe express CRH (**b**) and TH (**c**), but not DYN (**d**, shown by *). **B** **a** SCN in the human hypothalamus. PR**^+^** cells in the SCN did not express AVP (**b**) or DYN (**c**, shown by *). **C** **a** SDN in the human hypothalamus. PR**^+^** cells in the SDN did not express GAL (**b**, shown by *). Scale bars: 20 μm. Abbreviations: AVP, arginine vasopressin; CRH, corticotropin-releasing hormone; DYN, dynorphin; GAL, galanin; TH, tyrosine hydroxylase.


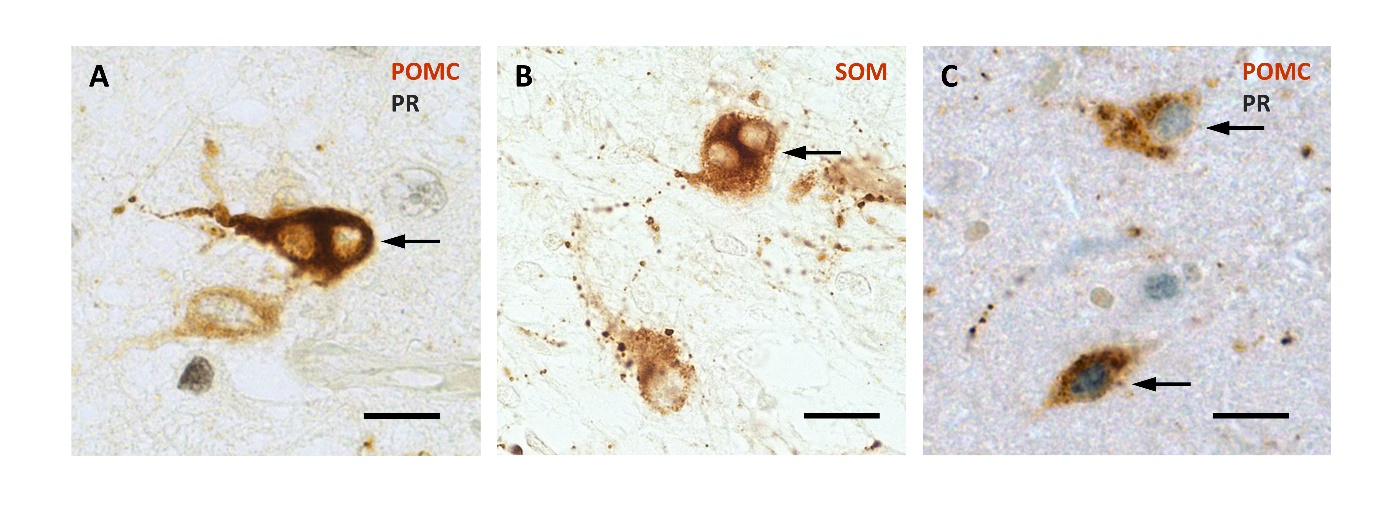


**Figure S3 POMC^+^ and SOM^+^ neurons in the infundibular nucleus. A** A bi-nuclear POMC**^+^** neuron (arrow). **B** A bi-nuclear SOM**^+^** neuron (arrow). **C** PR/POMC**^+^** neurons in the INF of a 4-year-old female (arrows). Scale bars: 20 μm. Abbreviations: POMC, pro-opiomelanocortin; PR/POMC, neurons co-labeling progesterone receptor and pro-opiomelanocortin; SOM, somatostatin.


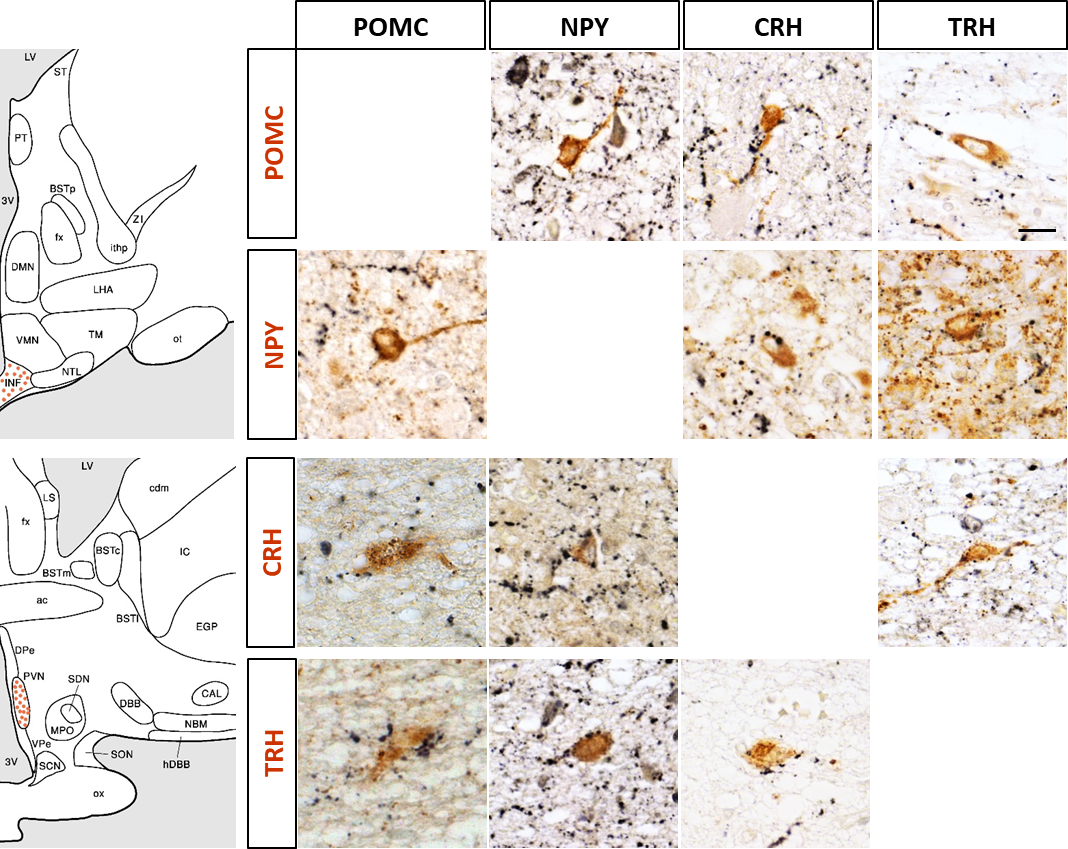


**Figure S4 Reciprocal innervation of POMC^+^, NPY^+^, CRH^+^ and TRH^+^ neurons in the infundibular nucleus and paraventricular nucleus.** Neurons are indicated in horizontal rows and in brown, fiber innervation is indicated in vertical columns and in black. Scale bar: 20 μm. Abbreviations: CRH, corticotropin-releasing hormone; NPY, neuropeptide Y; POMC, pro-opiomelanocortin; TRH, thyrotropin-releasing hormone.


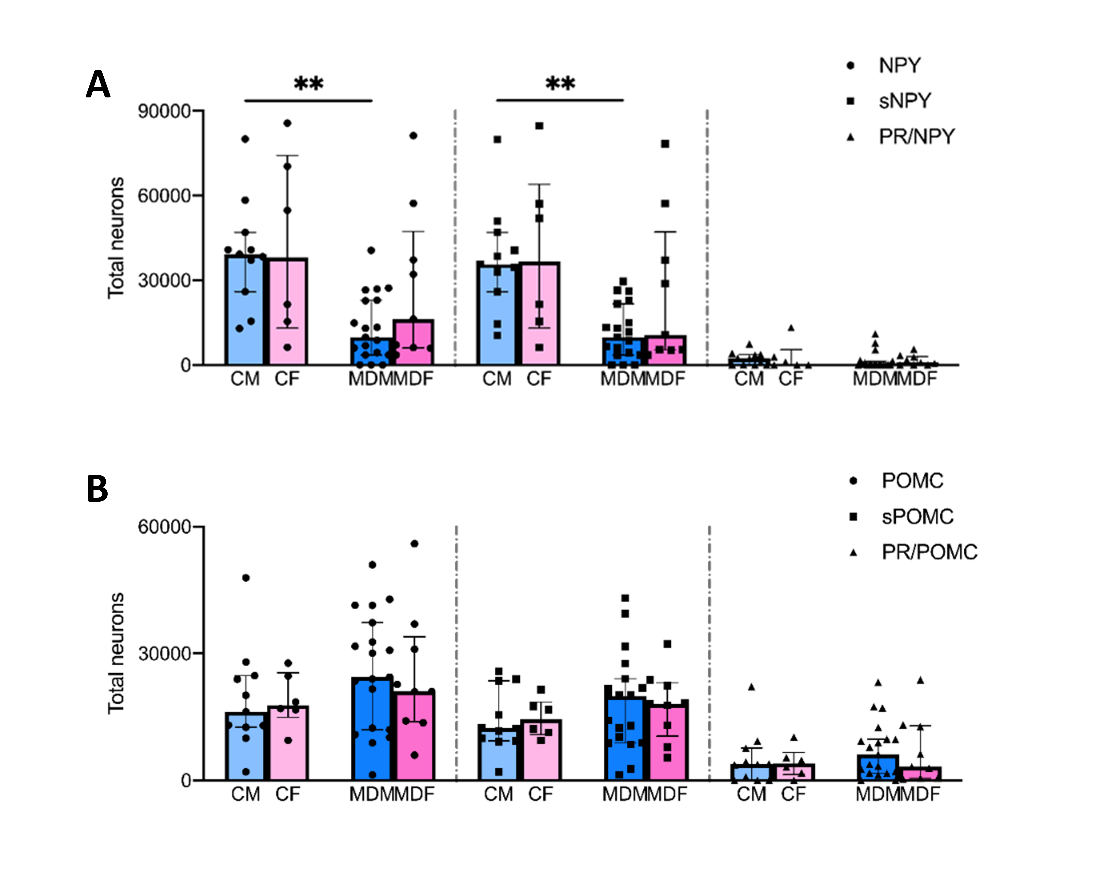


**Figure S5 Total number of NPY^+^ (a) and POMC^+^ (b) neurons and their subsets in the controls and patients with mood disorders between males and females.** Abbreviations: CF, control females; CM, control males; MDF, female patients with mood disorders; MDM, male patients with mood disorders; sNPY, NPY neurons that did not express PR; sPOMC, POMC neurons that did not express PR. Note: ** indicates 0.001≤*P*＜0.01.
