# Supplementary material for: Progesterone receptor distribution in the human hypothalamus and its association with suicide
Source: Acta Neuropathol Commun. 2024 Jan 23;12:16. doi: 10.1186/s40478-024-01733-y (PMC10807127; doi:10.1186/s40478-024-01733-y)
Supplement: Supplementary file 7 — Supplementary Material 7 [file 40478_2024_1733_MOESM7_ESM.docx]

Table S1a **Clinico-pathological information (PR mapping).**

| **Sample**  **number** | **Age** | **Age group** | **Sex** | **PMD (h)** | **CTD** | **CSF pH** | **BW (g)** | **Cause of death** |
| --- | --- | --- | --- | --- | --- | --- | --- | --- |
| CM1 | 40gw | Perinatal period | M | 14:05 | 19:55 | N.A. | 320 | Respiratory failure |
| CM2 | 4y | Childhood | M | 23:55 | 9:35 | N.A. | 1565 | Sepsis |
| CM3 | 17y | Adolescence | M | 41:00 | 0:00 | N.A. | 1499 | Myocarditis |
| CM4 | 27y | Youth | M | N.A. | N.A. | N.A. | N.A. | Drowning |
| CM5 | 55y | Middle age | M | 7:15 | 11:05 | N.A. | 1393 | Intestinal ischemia |
| CM6 | 79y | Elderly age | M | 6:30 | 10:00 | 6.71 | 1121 | Legal euthanasia |
| CF1 | 40gw | Perinatal period | F | 30:45 | 3:30 | N.A. | 380 | Asphyxia |
| CF2 | 7y | Childhood | F | 9:45 | 7:15 | N.A. | 1320 | Respiratory failure |
| CF3 | 15y | Adolescence | F | 20:00 | 13:00 | N.A. | 1480 | Lung edema |
| CF4 | 26y | Youth | F | 6:35 | 2:25 | N.A. | 1156 | Respiratory arrest |
| CF5 | 54y | Middle age | F | 5:35 | 15:15 | 6.40 | 1118 | Liver cirrhosis |
| CF6 | 83y | Elderly age | F | 3:20 | 16:30 | 6.80 | 1173 | Legal euthanasia |

**Abbreviations:** BW, brain weight; CF, control females; CM, control males; CSF, cerebral spinal fluid; CTD, clock time of death; F, female; g, gram; gw, gestational week; h, hour; M, male; N.A., not available; PMD, postmortem delay; y, year.

Table S1b **Clinico-pathological information (fetal hypothalami).**

| **Sample** | **Age (gw)** | **Sex** | **PMD (h)** | **CTD** | **BW (g)** | **Cause of death** |
| --- | --- | --- | --- | --- | --- | --- |
| 1 | 22 | M | 15:16 | 17:44 | N.A. | N.A. |
| 2 | 30 | M | 17:00 | 0:00 | 150 | Pneumonia |
| 3 | 37 | M | 17:00 | 0:00 | 400 | Congenital heart syndrome |

**Abbreviations:** BW, brain weight; CTD, clock time of death; g, gram; gw, gestational week; h, hour**;** M, male; N.A., not available; PMD, postmortem delay.

Table S1c **Clinico-pathological information (two-group comparison).**

| **Sample**  **number** | **Psychiatric diagnosis** | **Age (years)** | **Sex** | **PMD (h)** | **CTD** | **CSF pH** | **BW (g)** | **Suicide attempt** | **Antipsychotics in last three months** | **Cause of death** |
| --- | --- | --- | --- | --- | --- | --- | --- | --- | --- | --- |
| MD1 | MDD | 50 | M | N.A. | N.A. | N.A. | 1380 | Yes | N.A. | Suicide |
| MD2 | MDD | 39 | M | 41:00 | 00:00 | N.A. | 1670 | Yes | TCA, SSRI | Suicide |
| MD3 | BD | 39 | M | 48:00 | 11:30 | N.A. | 1220 | Yes | BZD, RIMA, SSRI | Suicide: intoxication |
| MD4 | MDD | 79 | M | 21:10 | 17:50 | N.A. | 1530 | Yes | BZD, SSRI | Suicide: high fall |
| MD5 | MDD | 74 | M | 62:55 | 17:05 | N.A. | 1444 | Yes | BZD, Cisordinol, SSRI | Suicide: hanging |
| MD6 | MDD | 62 | F | 20:35 | 00:00 | 6.53 | 1199 | Yes | BZD | Suicide: suffocation |
| MD7 | MDD | 56 | F | 10:30 | 14:00 | 6.42 | 1120 | Yes | BZD | Suicide: intoxication |
| MD8 | MDD | 62 | F | 11:40 | 16:15 | 6.18 | 1155 | Yes | BZD | Suicide: strangulation |
| MD9 | MDD | 58 | M | 4:30 | 9:40 | 6.80 | 1470 | Yes | SGA, SSRI, TCA | Suicide: intoxication |
| MD10 | MDD | 90 | F | 3:55 | 11:20 | 6.62 | 1220 | No | BZD, INN | Suicide: intoxication |
| MD11 | BD | 81 | M | 6:40 | 20:00 | 6.70 | 1283 | No | BZD, Li, TCA, VPA | Legal euthanasia |
| MD12 | MDD | 77 | F | 8:40 | 11:20 | 6.77 | 1120 | Yes | BZD, INN, SSRI | Legal euthanasia |
| MD13 | MDD | 50 | M | 9:40 | 13:00 | 6.80 | 1420 | No | BZD | Legal euthanasia |
| MD14 | MDD | 38 | M | 6:15 | 11:45 | 6.79 | 1525 | No | BZD | Legal euthanasia |
| MD15 | MDD | 61 | F | 8:40 | 15:35 | 7.07 | 1205 | No | BZD | Legal euthanasia |
| MD16 | MDD | 61 | M | 5:45 | 14:05 | N.A. | 1210 | No | SSRI | Legal euthanasia |
| MD17 | MDD | 67 | M | 3:30 | 11:10 | 6.72 | 1445 | No | BZD, Hal | Legal euthanasia |
| MD18 | MDD | 70 | M | 20:00 | 19:00 | N.A. | 1500 | No | BZD, DA stabilizer, SSRI | Heart attack |
| MD19 | MDD | 73 | F | 22:00 | 19:00 | N.A. | 1287 | No | BZD, TCA | Bronchopneumonia |
| MD20 | MDD | 71 | M | 13:30 | 22:30 | N.A. | 1109 | No | BZD, Li, MAOI, PTZ | Respiratory insufficiency |
| MD21 | MDD | 61 | M | 60:20 | 4:40 | N.A. | 1424 | No | PTZ | Pneumonia |
| MD22 | MDD | 81 | M | 6:00 | 15:30 | 6.50 | 1280 | No | Hal | Renal insufficiency |
| MD23 | MDD | 68 | M | 8:55 | 5:45 | 6.82 | 1510 | Yes | BZD, SSRI | Sudden death |
| MD24 | BD | 70 | M | 6:50 | 21:00 | 6.19 | 1275 | No | BZD, LTG, VPA | Pulmonary infection and renal insufficiency |
| MD25 | BD | 73 | M | 13:45 | 19:45 | 6.24 | 1480 | No | BZD | Infection |
| MD26 | BD | 94 | F | 5:10 | 4:00 | 7.55 | 985 | No | BZD, SSRI | Respiratory and cadiac failure |
| MD27 | MDD | 58 | F | 7:20 | 23:00 | 5.61 | 1295 | No | BZD, DA stabilizer, SSRI | Renal insufficiency |
| MD28 | BD | 65 | M | 4:50 | 18:30 | N.A. | 1305 | No | BZD, Li, SGA | Colon carcinoma |
| **Median** | - | 66 | - | 08:55 | 14:05 | 6.70 | 1291 | - | - | - |
| CTR1 | - | 68 | F | 5:45 | 12:15 | 6.97 | 1135 | No | None | Legal euthanasia |
| CTR2 | - | 83 | F | 3:20 | 16:30 | 6.80 | 1173 | No | None | Legal euthanasia |
| CTR3 | - | 49 | M | 6:15 | 17:30 | 6.15 | 1364 | No | BZD | Legal euthanasia |
| CTR4 | - | 79 | M | 6:30 | 10:00 | 6.71 | 1121 | No | BZD | Legal euthanasia |
| CTR5 | - | 83 | M | 5:45 | 19:35 | 7.28 | 1195 | No | None | Legal euthanasia |
| CTR6 | - | 41 | M | 17:00 | 00:00 | N.A. | 1150 | No | None | Renal insufficiency |
| CTR7 | - | 72 | F | 17:30 | 16:30 | N.A. | N.A. | No | None | Bronchopneumonia |
| CTR8 | - | 49 | M | 21:40 | 19:20 | N.A. | 1629 | No | None | Cardiac infarction |
| CTR9 | - | 74 | F | 7:25 | 9:50 | 6.95 | 1167 | No | BZD | Intestinal necrosis |
| CTR10 | - | 66 | M | 41:00 | 00:00 | N.A. | 1461 | No | INN | Septic shock |
| CTR11 | - | 88 | F | 5:55 | 7:00 | 6.05 | 1115 | No | BZD | Cardiac arrest |
| CTR12 | - | 39 | M | 16:30 | 00:30 | N.A. | 1400 | No | None | Cardiac infarction |
| CTR13 | - | 55 | M | 7:15 | 11:05 | N.A. | 1393 | No | BZD, INN | Intestinal ischemia |
| CTR14 | - | 78 | F | 4:35 | 22:40 | 6.41 | 1226 | No | BZD, INN | Bronchopneumonia |
| CTR15 | - | 73 | M | 8:00 | 16:15 | 5.37 | 1553 | No | BZD, INN | Pneumonia |
| CTR16 | - | 83 | M | 5:15 | 11:15 | 6.60 | 1372 | No | BZD | Cardiac infarction |
| CTR17 | - | 67 | M | 9:00 | 15:05 | 6.48 | 1292 | No | BZD, INN | Aortic aneurysm |
| **Median** | - | 72 | - | 07:15 | 12:15 | 6.60 | 1259 | - | - | - |
| ***P* value** | - | 0.45 | 0.83 | 0.34 | n.sig. | 0.75 | 0.47 | - | - | - |

**Abbreviations:** BD, bipolar disorder; BW, brain weight; BZD, benzodiazepine; CTD, clock time of death; CTR, control; DA, dopamine; F, female; Hal, haloperidol; INN, nemifitide; Li, lithium; LTG, lamotrigine; M, male; MAOI, monoamine oxidase inhibitor; MD, mood disorders; MDD, major depressive disorder; N.A., not available; PMD, postmortem delay; PTZ, pentylenetetrazole; RIMA, reversible inhibitor of monoamine oxidase-A; SGA, second-generation antipsychotics; SSRI, selective serotonin reuptake inhibitor; TCA, tricyclic antidepressant; VPA, valproate.

Table S1d **Clinico-pathological information (five-group comparison).**

| **Sample**  **number** | **Psychiatric diagnosis** | **Age (years)** | **Sex** | **PMD (h)** | **CTD** | **CSF pH** | **BW (g)** | **Suicide attempt** | **Antipsychotics in last three months** | **Cause of death** |
| --- | --- | --- | --- | --- | --- | --- | --- | --- | --- | --- |
| MDS1 | MDD | 50 | M | N.A. | N.A. | N.A. | 1380 | Yes | N.A. | Suicide |
| MDS2 | MDD | 39 | M | 41:00 | 00:00 | N.A. | 1670 | Yes | TCA, SSRI | Suicide |
| MDS3 | BD | 39 | M | 48:00 | 11:30 | N.A. | 1220 | Yes | BZD, RIMA, SSRI | Suicide: intoxication |
| MDS4 | MDD | 79 | M | 21:10 | 17:50 | N.A. | 1530 | Yes | BZD, SSRI | Suicide: high fall |
| MDS5 | MDD | 74 | M | 62:55 | 17:05 | N.A. | 1444 | Yes | BZD, Cisordinol, SSRI | Suicide: hanging |
| MDS6 | MDD | 62 | F | 20:35 | 00:00 | 6.53 | 1199 | Yes | BZD | Suicide: suffocation |
| MDS7 | MDD | 56 | F | 10:30 | 14:00 | 6.42 | 1120 | Yes | BZD | Suicide: intoxication |
| MDS8 | MDD | 62 | F | 11:40 | 16:15 | 6.18 | 1155 | Yes | BZD | Suicide: strangulation |
| MDS9 | MDD | 58 | M | 4:30 | 9:40 | 6.80 | 1470 | Yes | SGA, SSRI, TCA | Suicide: intoxication |
| MDS10 | MDD | 90 | F | 3:55 | 11:20 | 6.62 | 1220 | No | BZD, INN | Suicide: intoxication |
| **Median** | - | 60 | - | 20:35 | 11:30 | 6.53 | 1300 | - | - | - |
| MDE1 | BD | 81 | M | 6:40 | 20:00 | 6.70 | 1283 | No | BZD, Li, TCA, VPA | Legal euthanasia |
| MDE2 | MDD | 77 | F | 8:40 | 11:20 | 6.77 | 1120 | Yes | BZD, INN, SSRI | Legal euthanasia |
| MDE3 | MDD | 50 | M | 9:40 | 13:00 | 6.80 | 1420 | No | BZD | Legal euthanasia |
| MDE4 | MDD | 38 | M | 6:15 | 11:45 | 6.79 | 1525 | No | BZD | Legal euthanasia |
| MDE5 | MDD | 61 | F | 8:40 | 15:35 | 7.07 | 1205 | No | BZD | Legal euthanasia |
| MDE6 | MDD | 61 | M | 5:45 | 14:05 | N.A. | 1210 | No | SSRI | Legal euthanasia |
| MDE7 | MDD | 67 | M | 3:30 | 11:10 | 6.72 | 1445 | No | BZD, Hal | Legal euthanasia |
| **Median** | - | 61 | - | 6:40 | 13:00 | 6.78 | 1283 | - | - | - |
| MDN1 | MDD | 70 | M | 20:00 | 19:00 | N.A. | 1500 | No | BZD, DA stablizer, SSRI | Heart attack |
| MDN2 | MDD | 73 | F | 22:00 | 19:00 | N.A. | 1287 | No | BZD, TCA | Bronchopneumonia |
| MDN3 | MDD | 71 | M | 13:30 | 22:30 | N.A. | 1109 | No | BZD, Li, MAOI, PTZ | Respiratory insufficiency |
| MDN4 | MDD | 61 | M | 60:20 | 4:40 | N.A. | 1424 | No | PTZ | Pneumonia |
| MDN5 | MDD | 81 | M | 6:00 | 15:30 | 6.50 | 1280 | No | Hal | Renal insufficiency |
| MDN6 | MDD | 68 | M | 8:55 | 5:45 | 6.82 | 1510 | Yes | BZD, SSRI | Sudden death |
| MDN7 | BD | 70 | M | 6:50 | 21:00 | 6.19 | 1275 | No | BZD, LTG, VPA | Pulmonary infection and renal insufficiency |
| MDN8 | BD | 73 | M | 13:45 | 19:45 | 6.24 | 1480 | No | BZD | Infection |
| MDN9 | BD | 94 | F | 5:10 | 4:00 | 7.55 | 985 | No | BZD, SSRI | Respiratory and cadiac failure |
| MDN10 | MDD | 58 | F | 7:20 | 23:00 | 5.61 | 1295 | No | BZD, DA stabilizer, SSRI | Renal insufficiency |
| MDN11 | BD | 65 | M | 4:50 | 18:30 | N.A. | 1305 | No | BZD, Li, SGA | Colon carcinoma |
| **Median** | - | 70 | - | 8:55 | 19:00 | 6.37 | 1295 | - | - | - |
| CE1 | - | 68 | F | 5:45 | 12:15 | 6.97 | 1135 | No | None | Legal euthanasia |
| CE2 | - | 83 | F | 3:20 | 16:30 | 6.80 | 1173 | No | None | Legal euthanasia |
| CE3 | - | 49 | M | 6:15 | 17:30 | 6.15 | 1364 | No | BZD | Legal euthanasia |
| CE4 | - | 79 | M | 6:30 | 10:00 | 6.71 | 1121 | No | BZD | Legal euthanasia |
| CE5 | - | 83 | M | 5:45 | 19:35 | 7.28 | 1195 | No | None | Legal euthanasia |
| **Median** | - | 79 | - | 5:45 | 16:30 | 6.80 | 1173 | - | - | - |
| CN1 | - | 41 | M | 17:00 | 00:00 | N.A. | 1150 | No | None | Renal insufficiency |
| CN2 | - | 72 | F | 17:30 | 16:30 | N.A. | N.A. | No | None | Bronchopneumonia |
| CN3 | - | 49 | M | 21:40 | 19:20 | N.A. | 1629 | No | None | Cardiac infarction |
| CN4 | - | 74 | F | 7:25 | 9:50 | 6.95 | 1167 | No | BZD | Intestinal necrosis |
| CN5 | - | 66 | M | 41:00 | 00:00 | N.A. | 1461 | No | INN | Septic shock |
| CN6 | - | 88 | F | 5:55 | 7:00 | 6.05 | 1115 | No | BZD | Cardiac arrest |
| CN7 | - | 39 | M | 16:30 | 00:30 | N.A. | 1400 | No | None | Cardiac infarction |
| CN8 | - | 55 | M | 7:15 | 11:05 | N.A. | 1393 | No | BZD, INN | Intestinal ischemia |
| CN9 | - | 78 | F | 4:35 | 22:40 | 6.41 | 1226 | No | BZD, INN | Bronchopneumonia |
| CN10 | - | 73 | M | 8:00 | 16:15 | 5.37 | 1553 | No | BZD, INN | Pneumonia |
| CN11 | - | 83 | M | 5:15 | 11:15 | 6.60 | 1372 | No | BZD | Cardiac infarction |
| CN12 | - | 67 | M | 9:00 | 15:05 | 6.48 | 1292 | No | BZD, INN | Aortic aneurysm |
| **Median** | - | 70 | - | 8:30 | 11:10 | 6.45 | 1372 | - | - | - |
| ***P* value** | - | 0.43 | 0.97 | 0.07 | Sig. | 0.21 | 0.47 | - | - | - |

**Abbreviations:** BD, bipolar disorder; BW, brain weight; BZD, benzodiazepine; CTD, clock time of death; CTR, control; DA, dopamine; F, female; Hal, haloperidol; INN, nemifitide; Li, lithium; LTG, lamotrigine; M, male; MAOI, monoamine oxidase inhibitor; MD, mood disorders; MDD, major depressive disorder; N.A., not available; PMD, postmortem delay; PTZ, pentylenetetrazole; RIMA, reversible inhibitor of monoamine oxidase-A; SGA, second-generation antipsychotics; Sig., significance; SSRI, selective serotonin reuptake inhibitor; TCA, tricyclic antidepressant; VPA, valproate.

**Table S2 Specification of the antibodies used.**

| **Antibody** | **Species** | **Manufacturer** | **Catalog number and specificity** |
| --- | --- | --- | --- |
| PR | rabbit monoclonal | Abcam | ab32085 |
| α-MSH | rabbit polyclonal | NIN | α-MSH 4372 #23.04.75 ^1^ |
| NPY | rabbit polyclonal | NIN | Niepke #26.11.88 ^2^ |
| CRH | rat monoclonal | Gift NIN | PFU 83 ^3^ |
| TRH | rabbit polyclonal | Novus Biologicals | NBP2-34014 |
| GFAP | rabbit polyclonal | Agilent DAKO | GA524 |
| Iba1 | rabbit polyclonal | FUJIFILM Wako | 019-19741 |
| GFAP-δ | rabbit polyclonal | Gift NIN | 100501 ^4^ |
| Nestin | mouse monoclonal | Chemicon | MAB5326 |
| OXT | mouse monoclonal | Gift | OT-A-I-28 ^5^ |
| AVP | mouse monoclonal | Gift | VPIII-D-7 ^6^ |
| SOM | rabbit polyclonal | NIN | Somaar ^7^ |
| GAL | rabbit polyclonal | NIN | Gaaltje ^8^ |
| KISS1 | sheep polyclonal | Gift | GQ2 ^9^ |
| TH | mouse monoclonal | Sigma-Aldrich | MAB318 |
| DYN | rabbit polyclonal | Gift | Dynorphin (1-8) #73 ^10^ |

**Note:** 1 Specificities of commercial antibodies were provided by the manufacturers. The specificity of ab32085 refers to *Wagner, J., Rapsomaniki, M. A., Chevrier, S., Anzeneder, T., Langwieder, C., Dykgers, A., ... & Bodenmiller, B. (2019). A single-cell atlas of the tumor and immune ecosystem of human breast cancer. Cell, 177(5), 1330-1345*.

2 POMC-ir neurons were stained by α-MSH antibody ^11^.

Anti-CRH antibody is a kind gift from Prof. Dr. F.J.H. Tilders;

Anti-GFAP-δ antibody is a kind gift from Prof. Dr. E.M. Hol;

Anti-OXT antibody is a kind gift from Prof. Dr. F.W. van Leeuwen who obtained it from Dr. A. Hou Yu;

Anti-AVP antibody is a kind gift from Prof. Dr. F.W. Van Leeuwen who obtained it from Dr. A. Silverman;

Anti-KISS1 antibody is a kind gift from Prof. Dr. W.S. Dhillo;

Anti-DYN antibody is a kind gift from Prof. Dr. S.J. Watson.

**Abbreviations:** α-MSH, alpha-melanocyte-stimulating hormone; AVP, arginine vasopressin; CRH, corticotropin-releasing hormone; DYN, dynorphin; GAL, galanin; GFAP, glial fibrillary acidic protein; GFAP-δ, glial fibrillary acidic protein-delta; NIN, Netherlands Institute for Neuroscience; HLA-DP, DQ, DR, human leukocyte antigen-DP, DQ, DR isotypes; KISS1, kisspeptin; NPY, neuropeptide Y; OXT, oxytocin; PR, progesterone receptor; SOM, somatostatin; TH, tyrosine hydroxylase; TRH, thyrotropin-releasing hormone.

**References**

1. Swaab D, Visser M. A function for α-MSH in fetal development and the presence of an α-MSH-like compound in nervous tissue. *Melanocyte Stimulating Hormone: Control, Chemistry and Effects*, vol. 4. Karger Publishers1977, pp 170-178.

2. Fliers E, Unmehopa UA, Manniesing S, Vuijst CL, Wiersinga WM, Swaab DF. Decreased neuropeptide Y (NPY) expression in the infundibular nucleus of patients with nonthyroidal illness. *Peptides* 2001; **22**(3)**:** 459-465.

3. Raadsheer F, Sluiter A, Ravid R, Tilders F, Swaab D. Localization of corticotropin-releasing hormone (CRH) neurons in the paraventricular nucleus of the human hypothalamus; age-dependent colocalization with vasopressin. *Brain research* 1993; **615**(1)**:** 50-62.

4. Roelofs RF, Fischer DF, Houtman SH, Sluijs JA, Van Haren W, Van Leeuwen FW *et al.* Adult human subventricular, subgranular, and subpial zones contain astrocytes with a specialized intermediate filament cytoskeleton. *Glia* 2005; **52**(4)**:** 289-300.

5. Dai D, Li Q-C, Zhu Q-B, Hu S-H, Balesar R, Swaab D *et al.* Direct involvement of androgen receptor in oxytocin gene expression: possible relevance for mood disorders. *Neuropsychopharmacology* 2017; **42**(10)**:** 2064-2071.

6. Wu YH, Zhou JN, Balesar R, Unmehopa U, Bao A, Jockers R *et al.* Distribution of MT1 melatonin receptor immunoreactivity in the human hypothalamus and pituitary gland: colocalization of MT1 with vasopressin, oxytocin, and corticotropin‐releasing hormone. *Journal of Comparative Neurology* 2006; **499**(6)**:** 897-910.

7. Van de Nes J, Sluiter A, Pool C, Kamphorst W, Ravid R, Swaab D. The monoclonal antibody Alz-50, used to reveal cytoskeletal changes in Alzheimer's disease, also reacts with a large subpopulation of somatostatin neurons in the normal human hypothalamus and adjoining areas. *Brain research* 1994; **655**(1-2)**:** 97-109.

8. Garcia‐Falgueras A, Ligtenberg L, Kruijver FP, Swaab DF. Galanin neurons in the intermediate nucleus (InM) of the human hypothalamus in relation to sex, age, and gender identity. *Journal of Comparative Neurology* 2011; **519**(15)**:** 3061-3084.

9. Dhillo WS, Chaudhri OB, Patterson M, Thompson EL, Murphy KG, Badman MK *et al.* Kisspeptin-54 stimulates the hypothalamic-pituitary gonadal axis in human males. *The Journal of Clinical Endocrinology & Metabolism* 2005; **90**(12)**:** 6609-6615.

10. Sherman T, Day R, Civelli O, Douglass J, Herbert E, Akil H *et al.* Regulation of hypothalamic magnocellular neuropeptides and their mRNAs in the Brattleboro rat: coordinate responses to further osmotic challenge. *Journal of Neuroscience* 1988; **8**(10)**:** 3785-3796.

11. Goldstone AP, Unmehopa UA, Bloom SR, Swaab DF. Hypothalamic NPY and agouti-related protein are increased in human illness but not in Prader-Willi syndrome and other obese subjects. *The Journal of Clinical Endocrinology & Metabolism* 2002; **87**(2)**:** 927-937.

**Table S3a Immunohistochemistry for single staining.**

| **Antibody** | **Washing buffer** | **Antigen retrieval** | **Blocking buffer** | **Incubating buffer** | **Dilution** | **Incubating time** |
| --- | --- | --- | --- | --- | --- | --- |
| PR | 1×TBS | 0.01M citrate buffer  (pH 6.0)  microwave 800 w 20 min | - | SUMI ^1^ | 1:100 | 72 h |
| α-MSH | 3×TBS | 0.03M citrate buffer  (pH 6.0)  microwave 800 w 20 min | - | SUMI-HS ^2^ | 1:8000 | 24 h |
| NPY | 1×TBS | - | 1×TBS-5% milk | SUMI-milk ^3^ | 1:1000 | 24 h |

**Note:** 1 SUMI: supermix, 0.25 g gelatin in 100 ml 1×TBS, pH 7.6 heat the gelatin, mix until it dissolves, add Triton-X 0.5 ml. Store in fridge.

2 SUMI-HS: supermix-high salt, 0.25 g gelatin in 100 ml 3×TBS, pH 7.6 heat the gelatin, mix until it dissolves, add Triton-X 0.5 ml. Store in fridge.

3 SUMI-milk: add 5 g milk powder in 100 ml SUMI, mix until it dissolves. Prepare before use.

**Abbreviations:** α-MSH, alpha-melanocyte-stimulating hormone; NPY, neuropeptide Y; PR, progesterone receptor; TBS, tris-buffered saline.

**Table S3b Immunohistochemistry for double staining.**

| **Combo** | **Antibodies** | **Washing buffer** | **Antigen retrieval** | **Blocking buffer** | **Incubating buffer** | **Dilution** | **Incubating time** | **Color development** |
| --- | --- | --- | --- | --- | --- | --- | --- | --- |
| PR+α-MSH | PR | 1×TBS | 0.01M citrate buffer (pH 6.0)  microwave 800 w 20 min | - | SUMI | 1:100 | 72 h | DAB-Ni |
|  | α-MSH | 3×TBS | - | - | SUMI-HS | 1:4000 | 24 h | DAB |
| PR+NPY | PR | 1×TBS | 0.01M citrate buffer (pH 6.0)  microwave 800 w 20 min | - | SUMI | 1:100 | 72 h | DAB-Ni |
|  | NPY | 1×TBS | - | 1×TBS-5% milk | SUMI-milk | 1:500 | 24 h | DAB |
| PR+CRH | PR | 1×TBS | 0.01M citrate buffer (pH 6.0)  microwave 800 w 20 min | - | SUMI | 1:100 | 72 h | DAB-Ni |
|  | CRH | 1×TBS | - | - | SUMI | 1:50000 | 24 h | DAB |
| PR+TRH | PR | 1×TBS | 0.01M citrate buffer (pH 6.0)  microwave 800 w 20 min | - | SUMI | 1:100 | 72 h | DAB-Ni |
|  | TRH | 3×TBS | - | - | SUMI-HS | 1:2000 | 24 h | DAB |
| PR+GFAP | PR | 1×TBS | 0.01M citrate buffer (pH 6.0)  microwave 800 w 20 min | - | SUMI | 1:100 | 72 h | DAB-Ni |
|  | GFAP | 1×TBS | - | - | SUMI | 1:10000 | 24 h | DAB |
| PR+ iba1 | PR | 1×TBS | 0.01M citrate buffer (pH 6.0)  microwave 800 w 20 min | - | SUMI | 1:100 | 72 h | DAB-Ni |
|  | Iba1 | 1×TBS | - | - | SUMI | 1:500 | 24 h | DAB |
| PR+GFAP-δ | PR | 1×TBS | 0.01M citrate buffer (pH 6.0)  microwave 800 w 20 min | - | SUMI | 1:100 | 72 h | DAB-Ni |
|  | GFAP-δ | 1×TBS | - | - | SUMI | 1:250 | 24 h | DAB |
| PR+nestin | PR | 1×TBS | 0.01M citrate buffer (pH 6.0)  microwave 800 w 20 min | - | SUMI | 1:100 | 72 h | DAB-Ni |
|  | nestin | 1×TBS | - | - | SUMI | 1:200 | 24 h | DAB |
| PR+OXT | PR | 1×TBS | 0.01M citrate buffer (pH 6.0)  microwave 800 w 20 min | - | SUMI | 1:100 | 72 h | DAB-Ni |
|  | OXT | 1×TBS | - | - | SUMI | 1:500 | 24 h | DAB |
| PR+AVP | PR | 1×TBS | 0.05M Tris-HCl buffer (pH 9.0)  microwave 800 w 20 min | - | SUMI | 1:100 | 72 h | DAB-Ni |
|  | AVP | 1×TBS | - | - | SUMI | 1:100 | 24 h | DAB |
| PR+SOM | PR | 1×TBS | 0.05M Tris-HCl buffer (pH 9.0)  microwave 800 w 20 min | - | SUMI | 1:100 | 72 h | DAB-Ni |
|  | SOM | 3×TBS | - | - | SUMI-HS | 1:800 | 24 h | DAB |
| PR+GAL | PR | 1×TBS | 0.01M citrate buffer (pH 6.0)  microwave 800 w 20 min | - | SUMI | 1:100 | 72 h | DAB-Ni |
|  | GAL | 1×TBS | - | - | SUMI | 1:800 | 24 h | DAB |
| PR+KISS1 | PR | 1×TBS | 0.01M citrate buffer (pH 6.0)  microwave 800 w 20 min | - | SUMI | 1:100 | 72 h | DAB-Ni |
|  | KISS | - | - | - | SUMI | 1:50000 | 24 h | DAB |
| PR+TH | PR | 1×TBS | 0.05M Tris-HCl buffer (pH 9.0)  microwave 800 w 20 min | - | SUMI | 1:100 | 72 h | DAB-Ni |
|  | TH | 1×TBS | - | - | SUMI | 1:750 | 24 h | DAB |
| PR+DYN | PR | 1×TBS | 0.05M Tris-HCl buffer (pH 9.0)  microwave 800 w 20 min | - | SUMI | 1:100 | 72 h | DAB-Ni |
|  | DYN | 1×TBS | - | - | SUMI | 1:200 | 24 h | DAB |
| α-MSH+NPY | α-MSH | 3×TBS | 0.03M citrate buffer (pH 6.0)  microwave 800 w 20 min | - | SUMI-HS | 1:8000 | 24 h | DAB-Ni |
|  | NPY | 1×TBS | - | 1×TBS-5% milk | SUMI-milk | 1:500 | 24 h | DAB |
| α-MSH+CRH | α-MSH | 3×TBS | 0.03M citrate buffer (pH 6.0)  microwave 800 w 20 min | - | SUMI-HS | 1:8000 | 24 h | DAB-Ni |
|  | CRH | 1×TBS | - | - | SUMI | 1:50000 | 24 h | DAB |
| α-MSH+TRH | α-MSH | 3×TBS | 0.03M citrate buffer (pH 6.0)  microwave 800 w 20 min | - | SUMI-HS | 1:8000 | 24 h | DAB-Ni |
|  | TRH | 3×TBS | - | - | SUMI-HS | 1:2000 | 24 h | DAB |
| NPY+α-MSH | NPY | 1×TBS | 0.01M citrate buffer (pH 6.0)  microwave 800 w 20 min | 1×TBS-5% milk | SUMI-milk | 1:1000 | 24 h | DAB-Ni |
|  | α-MSH | 3×TBS | - | - | SUMI-HS | 1:4000 | 24 h | DAB |
| NPY+CRH | NPY | 1×TBS | - | 1×TBS-5% milk | SUMI-milk | 1:1000 | 24 h | DAB-Ni |
|  | CRH | 1×TBS | - | - | SUMI | 1:50000 | 24 h | DAB |
| NPY+TRH | NPY | 1×TBS | 0.01M citrate buffer (pH 6.0)  microwave 800 w 20 min | 1×TBS-5% milk | SUMI-milk | 1:1000 | 24 h | DAB-Ni |
|  | TRH | 3×TBS | - | - | SUMI-HS | 1:2000 | 24 h | DAB |
| CRH+α-MSH | CRH | 1×TBS | 0.01M citrate buffer (pH 6.0)  microwave 800 w 20 min | - | SUMI | 1:100000 | 24 h | DAB-Ni |
|  | α-MSH | 3×TBS | - | - | SUMI-HS | 1:4000 | 24 h | DAB |
| CRH+NPY | CRH | 1×TBS | - | - | SUMI | 1:100000 | 24 h | DAB-Ni |
|  | NPY | 1×TBS | - | 1×TBS-5% milk | SUMI-milk | 1:500 | 24 h | DAB |
| CRH+TRH | CRH | 1×TBS | 0.01M citrate buffer (pH 6.0)  microwave 800 w 20 min | - | SUMI | 1:100000 | 24 h | DAB-Ni |
|  | TRH | 3×TBS | - | - | SUMI-HS | 1:2000 | 24 h | DAB |
| TRH+α-MSH | TRH | 3×TBS | 0.03M citrate buffer (pH 6.0)  microwave 800 w 20 min | - | SUMI-HS | 1:4000 | 24 h | DAB-Ni |
|  | α-MSH | 3×TBS | - | - | SUMI-HS | 1:4000 | 24 h | DAB |
| TRH+NPY | TRH | 3×TBS | 0.03M citrate buffer (pH 6.0)  microwave 800 w 20 min | - | SUMI-HS | 1:4000 | 24 h | DAB-Ni |
|  | NPY | 1×TBS | - | 1×TBS-5% milk | SUMI-milk | 1:500 | 24 h | DAB |
| TRH+CRH | TRH | 3×TBS | 0.03M citrate buffer (pH 6.0)  microwave 800 w 20 min | - | SUMI-HS | 1:4000 | 24 h | DAB-Ni |
|  | CRH | 1×TBS | - | - | SUMI | 1:50000 | 24 h | DAB |

**Note:** 1 SUMI: supermix, 0.25 g gelatin in 100 ml 1×TBS, pH 7.6 heat the gelatin, mix until it dissolves, add Triton-X 0.5 ml. Store in fridge.

2 SUMI-HS: supermix-high salt, 0.25 g gelatin in 100 ml 3×TBS, pH 7.6 heat the gelatin, mix until it dissolves, add Triton-X 0.5 ml. Store in fridge.

3 SUMI-milk: add 5 g milk powder in 100 ml SUMI, mix until it dissolves. Prepare before use.

**Abbreviations:** α-MSH, alpha-melanocyte-stimulating hormone; AVP, arginine vasopressin; CRH, corticotropin-releasing hormone; DAB, 3,3'-diaminobenzidine; DAB-Ni, 3,3'-diaminobenzidine-nickel; DYN, dynorphin; GAL, galanin; GFAP, glial fibrillary acidic protein; GFAP-δ, glial fibrillary acidic protein-delta; HLA-DP, DQ, DR, human leukocyte antigen-DP, DQ, DR isotypes; KISS1, kisspeptin; NPY, neuropeptide Y; OXT, oxytocin; PR, progesterone receptor; SOM, somatostatin; TBS, tris-buffered saline; TRH, thyrotropin-releasing hormone.
